# Healthy human induced pluripotent stem cell-derived cardiomyocytes exhibit sex dimorphism even without the addition of hormones

**DOI:** 10.1093/stmcls/sxaf038

**Published:** 2025-06-20

**Authors:** Sophie E Givens, Abygail A Andebrhan, Eric G Schmuck, Aimee Renaud, An Xie, Somayeh Ebrahimi-Barough, Juan E Abrahante, Noah Stanis, Samuel Dudley, James R Dutton, Brenda M Ogle

**Affiliations:** Biomedical Engineering, University of Minnesota, Minneapolis, MN 55455, United States; Biomedical Engineering, University of Minnesota, Minneapolis, MN 55455, United States; Stem Cell & Regenerative Medicine Center, University of Wisconsin-Madison, Madison, WI 53705, United States; Stem Cell Institute, University of Minnesota, Minneapolis, MN 55455, United States; Lillehei Heart Institute, University of Minnesota, Minneapolis, MN 55455, United States; Biomedical Engineering, University of Minnesota, Minneapolis, MN 55455, United States; Informatics Institute, University of Minnesota, Minneapolis, MN 55455, United States; Biomedical Engineering, University of Minnesota, Minneapolis, MN 55455, United States; Lillehei Heart Institute, University of Minnesota, Minneapolis, MN 55455, United States; Stem Cell Institute, University of Minnesota, Minneapolis, MN 55455, United States; Institute of Engineering in Medicine, University of Minnesota, Minneapolis, MN 55455, United States; Department of Genetics, Cell Biology and Development, University of Minnesota, Minneapolis, MN 55455, United States; Biomedical Engineering, University of Minnesota, Minneapolis, MN 55455, United States; Stem Cell Institute, University of Minnesota, Minneapolis, MN 55455, United States; Institute of Engineering in Medicine, University of Minnesota, Minneapolis, MN 55455, United States; Department of Pediatrics, University of Minnesota, Minneapolis, MN 55455, United States

**Keywords:** adhesion receptors, calcium flux, cardiac, induced pluripotent stem cells, iPS

## Abstract

Human induced pluripotent stem cell-derived cardiomyocytes (hiPSC-CMs) are a valuable cell type for studying human cardiac health and disease in vitro. However, it is not known whether hiPSC-CMs display sex dimorphism and therefore whether sex should be incorporated as a biological variable in in vitro studies that include this cell type. To date, the vast majority of studies that utilize hiPSC-CMs do not include both male and female sex nor stratify results based on sex because it is challenging to amass such a cohort of cells. Here, we generated 3 female and 3 male hiPSC lines from adult left ventricular cardiac fibroblasts as a resource for studying sex differences in in vitro cardiac models. We used this resource to generate hiPSC-CMs and maintained them in basal media without exogenous hormones. Functional assessment of CMs showed enhanced calcium handling in female-derived hiPSC-CMs relative to male. Bulk RNA sequencing revealed over 300 differentially expressed genes (DEGs) between male and female hiPSC-CMs. Gene ontology analysis of DEGs showed distinct differences in pathways related to cardiac pathology including cell-cell adhesion, metabolic processes, and response to ischemic stress. Differential expression of the sodium channel auxiliary unit *SCN3B* was found and validated through patch-clamp measurements of sodium currents, showing increased peak amplitude and window current in female hiPSC-CMs. These findings highlight the importance of considering sex as a variable when conducting studies to evaluate aspects of human cardiac health and disease related to CM function.

## Graphical Abstract

**Graphical Abstract:**
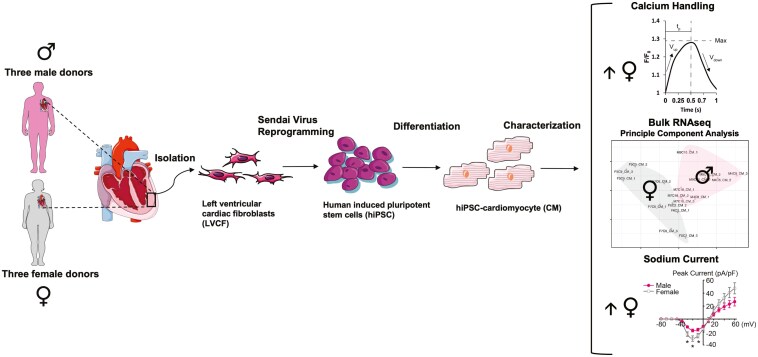

**Significance Statement**

This paper is novel as sex differences have never been analyzed in human induced pluripotent cardiomyocyte bulk RNA sequencing and/or calcium handling. Even without the addition of exogenous hormones, there were significant differences detected in both calcium handling and in bulk RNA sequencing.

## Introduction

Cardiovascular disease (CVD) is the leading cause of mortality and morbidity globally,^1^ but historically it has been challenging to study *human* CVD at the cellular level. Over the past decade, stem cells, particularly human induced pluripotent stem cells (hiPSCs), have become a source for creating in vitro model systems to study *human* CVD. Robust protocols for yielding heart muscle cells or cardiomyocytes (CMs) have propelled this approach.^2-4^ However, the use of this model system rarely considers sex as a biological variable though sex is a key mediator of CVD onset, progression, and prognosis in the clinic.

When developing models of CVD, consideration of sex dimorphism is essential as clinical and animal studies have revealed stark sex differences in CVD onset and progression.^5-8^ For example, women with ischemic heart disease (IHD) often have microvasculature and endothelial dysfunction and are more prone to spontaneous coronary artery dissection.^9,10^ In contrast, men experience higher rates of coronary artery dissections linked to IHD and are more susceptible to ischemia-reperfusion injury.^9,11,12^ Additionally, studies have shown distinct differences in myocardial response to pressure overload, a common precursor of heart failure (HF), in female and male animals.^13-15^ HF itself also shows a sex-dependent divergence; HF with preserved ejection fraction, coupled with increased ventricular stiffness, is more common in women, whereas men are more afflicted by HF with reduced ejection fraction, leading to ventricular dilation.^5^ Arrhythmias demonstrate similar sex-specific occurrences. Women have higher incidence of acquired long-QT syndrome and Torsades de Pointes, while men are at increased risk for atrial fibrillation and sudden cardiac arrest.^6^ Most sex differences detected in CVD thus far have been attributed to genetic, epigenetic, or hormonal factors.^7,8^ Since these factors can be hard to uncouple, especially in a human system and at the cellular level, there is still much to learn about sex dimorphism in human CVD. Given limited access to human cells and tissues, hiPSCs are an enabling tool to study human sex dimorphism in the cardiovascular system.

Numerous models of CVD have been generated using hiPSC-derived CMs (hiPSC-CMs) since their first derivation more than a decade ago.^16,17^ These include disease models of IHD and ischemia-reperfusion injury^18-20^ and even more commonly studies of cardiac arrhythmias are done using hiPSC-CMs; these include hiPSC-CM models of long-QT, Torsades de Pointes, and atrial fibrillation, all of which have demonstrated sex dimorphism in clinical studies.^21-25^ Interestingly, there has been very little consideration of sex dimorphism in these models. There have only been a few studies utilizing stem cell-derived CMs that look at sex differences. Among them, one study looked at sex differences in human embryonic stem cell-derived CM hypertrophy in response to isoproterenol using one female and one male line. Results showed that female CMs undergo hypertrophy more slowly in response to isoproterenol, indicating cellular-level differences in CM hypertrophy without the addition of hormones.^26^ In another study, 3 female and 3 male hiPSC-CM lines from different ethnic origins were tested for sensitivity to various pharmacological agents.^27^ This study found that hiPSC-CMs from females were more sensitive to rapid delayed rectifier potassium channel (I_Kr_) blockers than hiPSC-CMs from males. This finding was confirmed in another study using hiPSC-CMs to study drug-induced Torsades de Pointes; in this study, female hiPSC-CMs exhibited an increase in long-QT generation when exposed to I_Kr_ blockers.^28^ The exogenous addition of hormones affected hiPSC-CM electrophysiology but did not change the sensitivity of the female hiPSC-CMs to I_Kr_ blockers. This was attributed to increased expression of KCNE1, a critical cardiac potassium channel regulatory subunit, in male hiPSC-CMs. These studies were significant as they showed that the potential mechanism for sex dimorphism in drug-acquired Torsades de Pointes is not hormone-mediated. These studies support the possibility that other cellular differences in human CMs are present without hormones which are commonly thought to be the major mediator of sex dimorphism in CVD. The few studies that look at sex differences in hiPSC-CM model systems motivate future work, wherein hiPSC-CMs are used to determine the mechanism of CVD sex dimorphism in a controlled manner.

Here, we developed a cohort of male and female hiPSC lines from 6 healthy donors as a resource to enable the study of sex dimorphism with cardiac health and disease. The hiPSCs were made from the same cell source, at a similar donor age, and with the same reprogramming methodology to control for epigenetic noise.^29,30^ Though other larger hiPSC resources exist, many still cobble together lines from multiple cell sources that were derived using varying methodologies in multiple labs. Additionally, these lines were made from cardiac fibroblast as they can yield more functionally relevant hiPSC-CMs.^31-33^ To better understand if there are sex differences in hiPSC-CMs without the exogenous addition of hormones, we differentiated hiPSC-CMs from all of the hiPSC lines generated and maintained them in basal CM media (RPMI/B27 with insulin). We opted to conduct our comparison without the addition of sex hormones to mimic the setting of most basic bench research utilizing hiPSC-CMs and to uncouple hormone regulation from basal function of hiPSC-CMs.

## Materials and methods

Briefly, primary human aLVCFs from male and female donors were reprogrammed into hiPSCs using the Sendai virus reprogramming kit Cyto-tune 2.0 from Thermo Fisher Scientific. The hiPSCs were differentiated into cardiomyocytes using small molecule WNT modulation and were assessed for purity via flow cytometry, for cardiac functionality using calcium transients and patch clamp, and for gene expression using bulk RNA sequencing. See Supplementary Material for detailed explanation of the materials and methods.

## Results

### Sex is underreported in studies that utilize hiPSC-CMs

To determine whether sex has been considered in hiPSC-CM research, we looked at literature over the past 13 years, starting in 2010 when hiPSC-CM differentiation protocols first began to appear. We searched for all papers pertaining to hiPSC-CMs, with the query “(Human Induced Pluripotent Stem Cell derived Cardiomyocytes) OR hiPSC-CM OR (human iPSC-CMs).” As expected, the number of papers drastically increased from 2010 to 2023 (**Figure S1A**). This search was refined for any papers mentioning hiPSC-CMs and sex, male, or female to determine if one of these terms was even mentioned in the manuscripts. Only around 20% of the papers mentioning hiPSC-CMs also mentioned the terms “sex,” “male,” or “female” (**Figure S1B**). Further narrowing our focus, we determined that only 1% of papers about hiPSC-CMs mentioned “sex,” “male,” and “female” at the same time (**Figure S1C**), indicating it is likely that less than 1% of the hiPSC-CM literature uses both male and female lines or stratifies results by sex. To validate this, the top 50 hiPSC-CM primary research papers between the year of 2010 and 2023, if there were 50 papers for that year, on PubMed were analyzed more closely (**Figure S1D**). The papers were probed carefully to determine if (1) the sex of the hiPSC line or lines used in the study were reported and (2) if the results were analyzed by sex. We observed a gradual increase in the percentage of papers reporting the sex of cell lines over this period (**Figure S1E**). Despite this increase, by 2022, only 70% of the papers reported the sex of the lines they used or reported the line origin in which the sex could be easily determined. Additionally, only 3 papers in this search analyzed their results by sex (**Figure S1F**). This suggests that studies rarely compared their findings between hiPSC-CMs from both male and female donors. In summary, more papers are reporting the sex of hiPSC-CM utilized, but sex-related differences in these studies are not being evaluated.

### Creating a cohort of male and female hiPSCs with consistent cell source and reprogramming method

To enable studies of human CM that allow for the stratification of results according to sex, human adult left ventricular fibroblasts from 3 female and 3 male lines were reprogrammed into hiPSCs using the Sendai virus (SeV) (**Figure S2A**). Young adult donors between the ages of 21 and 34 years with no known cardiac abnormalities were used (**Table 1 and** Table S1). All donors were White, and no detailed ethnicity information was obtained (**Table S1**). Three lines were generated from individual colonies chosen from each donor, and all 18 lines were confirmed to be SeV depleted via qRT-PCR (**Table 1 and** Table S2). One line from each donor was assessed for pluripotency using qRT-PCR for the pluripotency genes OCT4, SOX2, and NANOG in comparison to a previously established hiPSC line, the CCND2-hiPSC line (**Figure S2B**).^34^ Representative brightfield images of line 1 (L1) from all 6 donors show the distinct colony morphology characteristic of hiPSCs (**Figure S2C**). The gene expression of *OCT4* was validated at the protein level using flow cytometry showing >95% Oct4^+^ cells in L1 from all 6 donors (**Figure S2D**). The cell surface pluripotency marker SSEA4 was also present in all 6 cell lines (**Figure S2E**). The pluripotency markers Sox2 and TRA-1-60 were present in the F6, F7, M4, and M7 lines, as detected by immunohistochemistry (**Figure S3A**). Similarly, coexpression of the transcription factor Oct4 was detected together with the cell surface marker SSEA4 (**Figure S3B**).

**Table 1.** Adult left ventricular fibroblasts line donor age, line SeV depletion, and karyotype.

Line name	Sex (F/M)	Age (years)	Karyotype	SeV depletion (passage #)
F5L1	F	26	Normal XX (QC)	18
F6L1	F	34	Normal XX (QC)	16
F7L1	F	36	Normal XX (Full)	12
M4L1	M	21	Normal XY (Full)	12
M7L1	M	21	Normal XY (QC)	8
M9L10	M	27	Normal XY (QC)	8

To ensure that no chromosomal abnormalities were acquired during SeV reprogramming, karyotype analysis was performed for 20 metaphases of one male (M4L1) and one female (F7L1) line (**Figure S4**). Quality control karyotype analysis examining 7 cells per line with the readout of “normal” or “abnormal” was conducted for L1 of the remaining donors. Karyotyping revealed a normal “XX” karyotype for all 3 female lines and a normal “XY” karyotype for all 3 male lines (**Table 1** and Table S2). Next, L1 from all 6 donors were differentiated into hiPSC-CMs to evaluate genetic and functional characteristics.

### Female hiPSC-CMs exhibit net enhanced calcium transient properties

**Figure 1.**
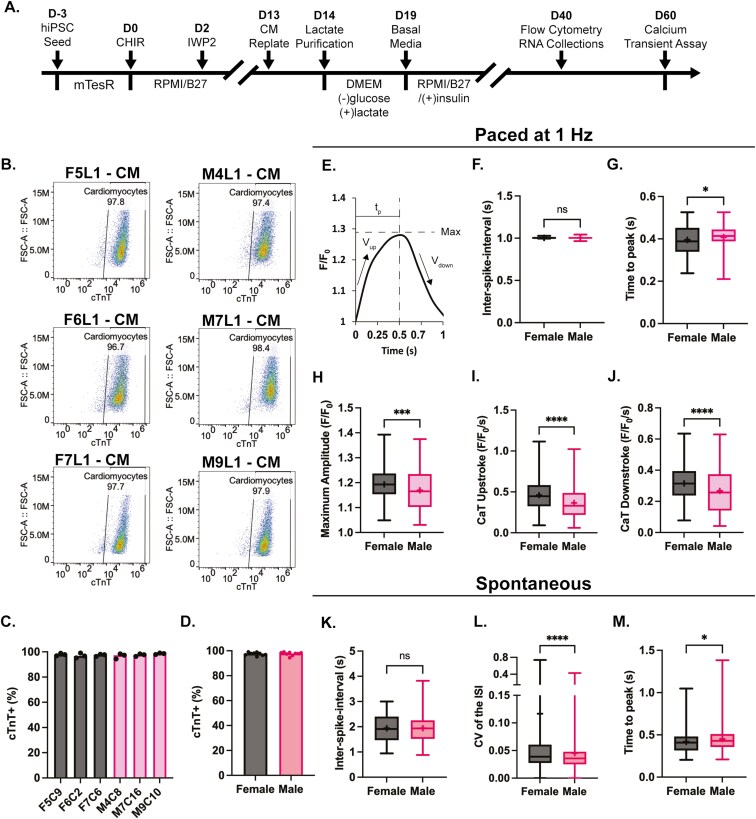
Female human induced pluripotent stem cell-derived cardiomyocytes (hiPSC-CMs) have enhanced calcium-handling properties. (A) Schematic depicting the hiPSC-CM differentiation, purification, and the timepoints of CM purity assessment, RNA collection, patch-clamp, and calcium-handling data acquisition. (B) Representative flow cytometry data showing the cells on forward scatter (FSC) vs CM marker, cardiac troponin T (cTnT) on the x-axis. (C) Quantification of the percent CMs (cTnT+) by line and (D) by sex where the bar represents the average between *n* = 3 independent experiments across all 6 lines. Each dot represents the given cTnT+ (%) for one replicate. (E) Representative paced calcium handling trace (from M4L1-CM) showing some of the parameters assessed, such as the time to peak (t_p_), the maximum amplitude (Max), and the maximum upstroke (V_up_) and downstroke (V_down_) velocities. Calcium transient (CaT) of the male and female hiPSC-CM bulk monolayers showing the (F) inter-spike interval (ISI), (G) time to peak, (H) maximum amplitude, and the CaT (I) upstroke and (J) downstroke. Box and whisker plots of the spontaneous CaT parameters, the (K) ISI, (L) the coefficient of variance (CV) of the ISI, showing the regularity of hiPSC-CM spontaneous CaT, and the (M) time to peak, for the male and female hiPSC-CMs. For (F-M), the box and whisker plots represent the median, upper, and lower quartiles while the (+) represents the mean. (F-M) The data are the average of 9 videos across 3 technical replicates for 3 independent experiments from all 3 female and all 3 male hiPSCs. Statistical comparisons were made using a pairwise Student’s *t*-test with *P < .05, **P < .01, ***P < .001, and ****P < .0001.

The female and male hiPSC lines were differentiated into CMs using small molecule WNT modulation and purified using lactate metabolic selection media (**Figure 1A**).^2,35^ On day 40, CM purity was assessed via flow cytometry for the CM-specific marker cardiac troponin T (cTnT) (**Figure 1B**). CMs displayed high purity for all 6 cell lines, with more than 95% of cells positive for cTnT (**Figure 1C**). The hiPSC-CM purity was not significantly different when analyzed by sex (**Figure 1D**).

On day 60, calcium functional analysis was performed on hiPSC-CM 2D monolayers. The calcium transient (CaT) inter-spike interval (ISI), time to peak, maximum amplitude, as well as the downstroke and upstroke velocities were assessed (**Figure 1E**). CM monolayers were analyzed under both paced and spontaneous conditions. For the paced conditions, the hiPSC-CMs were electrically stimulated at a frequency of 1 Hz and had an ISI of 1 (**Figure 1F**). The CaT time to peak is slightly but significantly decreased in the female hiPSC-CMs (**Figure 1G**), indicating a faster net release of calcium from the sarcoplasmic reticulum of female hiPSC-CMs. Additionally, the CaT maximum amplitude as well as the CaT downstroke and upstroke velocities were significantly higher in female CMs (**Figure 1**H-J). Though there is a net increase in the female hiPSC-CM calcium handling when all of the lines are averaged, there is substantial donor-to-donor variability (**Figure S5A-E**). The spontaneous CaT activity was assessed to determine if there were any intrinsic differences in CM beat rate or the regularity of beating as assessed by the coefficient of variance (CV) of the ISI. No significant differences in the spontaneous ISI were observed between the female and male hiPSC-CMs (**Figure 1K**). However, the CV of the ISI was significantly greater in the female lines, indicating that the beat rate is more irregular in female hiPSC-CMs compared to males (**Figure 1L**). Additionally, the decrease in time to peak in the female hiPSC-CMs was seen in the spontaneous case as well (**Figure 1M**). The spontaneous calcium data also showed significant line-to-line variability (**Figure S3F-H**). These results show a net increase in the calcium-handling ability of female hiPSC-CMs in combination with a tendency for more irregular spontaneous beating frequencies.

### Bulk RNA sequencing of hiPSC-CMs reveals distinct hierarchical clustering according to sex

**Figure 2.**
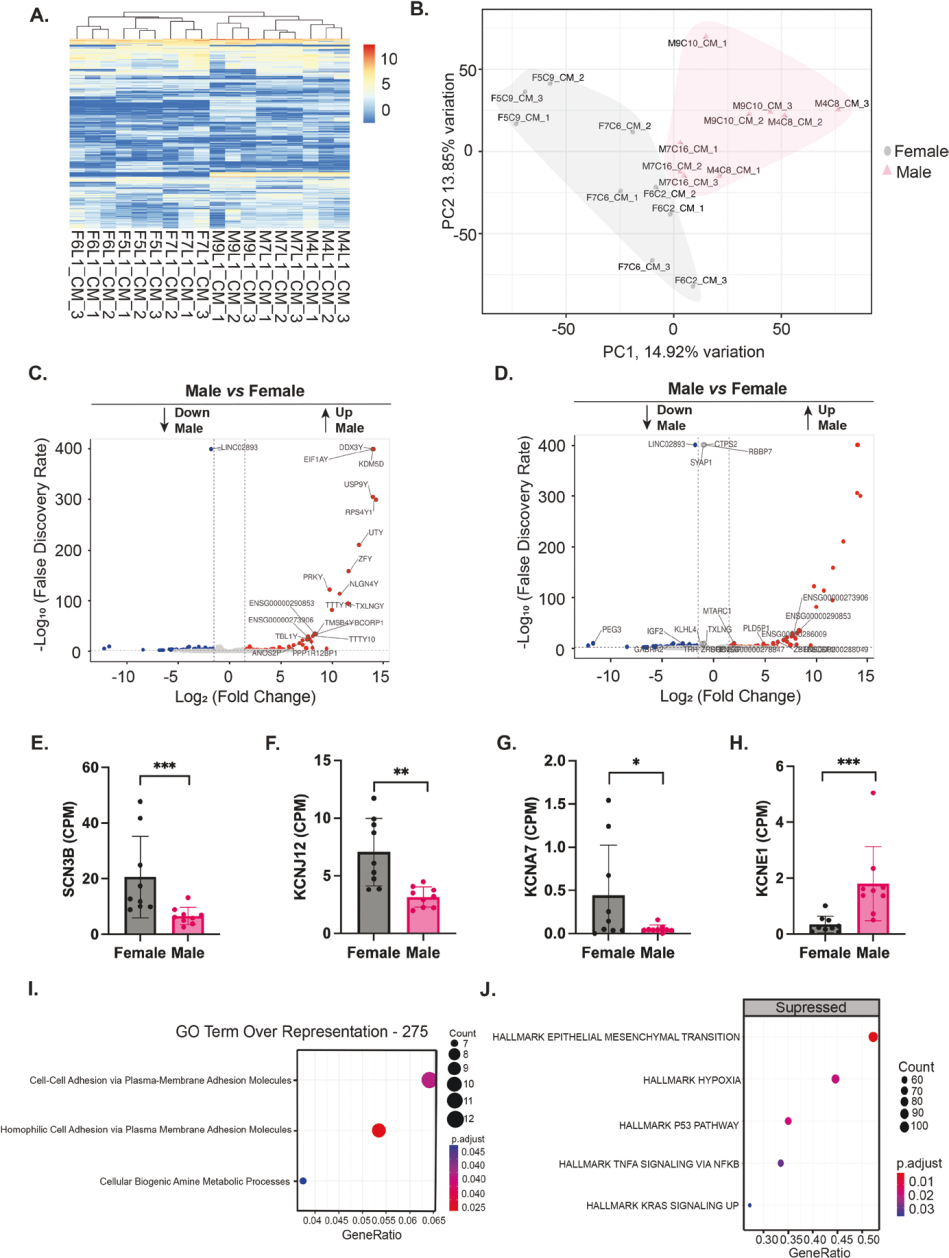
Bulk RNA sequencing reveals genetic differences between female and male human induced pluripotent stem cell-derived cardiomyocytes (hiPSC-CMs). (A) Heat map of hiPSC-CM gene expression from 3 independent experiments for all 6 hiPSC lines. A dendrogram at the top of the heatmap shows separate hierarchical clustering of male and female hiPSC-CMs based on gene expression patterns. (B) Principal component analysis (PCA) plot showing separate clustering of male and female hiPSC-CMs, where each dot is one independent RNA sequencing replicate. (C) Volcano plot representing differentially expressed genes (DEGs) between male and female hiPSC-CMs. (D) Volcano plot analogous to (C) with non-Y-linked genes highlighted on the volcano plot. (E) Dot plot showing enriched gene ontology (GO) biological process (BP) pathways from an overrepresentation analysis of the DEGs between the male and female hiPSC-CMs. The dot size indicates the number of genes linked to each enriched pathway, while the dot color reflects the statistical significance of the enrichment. (F) Hallmark gene set enrichment dot plot highlighting gene sets that are suppressed in male hiPSC-CMs compared to the female hiPSC-CMs. The dot size signifies the number of genes associated with enrichment, and the dot color denotes the level of statistical significance.

On the same day 40, RNA was collected from all the male and female hiPSC-CMs for bulk RNA sequencing. Three independent experiments from each hiPSC line were analyzed. A heat map generated from the expression profiles of all 18 samples displayed a distinct hierarchical clustering between male (M) and female (F) hiPSC-CMs (**Figure 2A**). Principal component analysis (PCA) showed distinct clustering of the male and female hiPSC-CMs (**Figure 2B**).

Differentially expressed genes (DEGs) between the female and male hiPSC-CMs were determined by grouping all the samples from each line together by sex. There were 347 DEGs with a false discovery rate (FDR) corrected *P*-value <.05 (**Supplementary File 1**). These were plotted as a volcano plot with the genes upregulated in female hiPSC-CMs and downregulated in male hiPSC-CMs having a negative fold change (**Figure 2C**). A volcano plot with the top 20 non-Y-linked DEGs in the male and female-derived CMs is also displayed (**Figure 2D**). Of the 347 genes differentially expressed, 13 of them were Y-linked, 14 of them were X-linked, and all others were genes located on the autosomes (**Figure S6**). Next, DEGs were examined in an unbiased manner using gene ontology (GO).

### GO pathways enrichment reveals sex differences in CM cell adhesion and amine metabolism

To better understand the differences in gene expression between male and female hiPSC-CMs, GO overrepresentation analysis was used to find out if any pathways were enriched between the female and male hiPSC-CMs. Both “Cell-Cell Adhesion via Plasma-Membrane Adhesion Molecules” and “Homophilic Cell Adhesion via Plasma Membrane Adhesion Molecules” were enriched GO biological process (BP) pathways, processes that are crucial for cell interaction and communication (**Figure 2E**). Additionally, the pathway “cellular biogenic amine metabolic process” was also enriched. Using a computer network plot (cnet) we visualized the connections between specific genes that were responsible for the GO term enrichment of these pathways (**Figures S7****and****S8**). To determine which genes were up- and downregulated, bar plots of the normalized counts per million (CPM) of all the significantly enriched genes in each pathway were plotted. We found that the hiPSC-CMs had differential expression of multiple cadherins (CDHs). Female hiPSC-CMs had increased expression of *CDH1* and *CDH17*, while male cells showed increased expression of *CDH7* and *CHD12* (**Figure S7B****-****M**). These results indicate that male and female hiPSC-CMs potentially adhere to each other by using different proteins even in the absence of exogenous hormones. Thus, differences in cell-cell adhesion may be mediators of non-hormone-mediated sex dimorphism in cardiac health and disease.

When looking at DEGs for the amine metabolism pathway, a large and distinct increase in thyrotropin-releasing hormone (*TRH*) was revealed in all the replicates and lines for the female hiPSC-CMs (**Figure S8B**). TRH is a cardiac hormone that has important functions in heart regulation and the responses to cardiac stress. It is induced after myocardial infarction and serves as a positive inotrope, increasing cardiac contractility and output, but long-term activation can lead to a hypertrophic phenotype in the heart.^36-39^ Two gene transcripts of interest, one upregulated and one downregulated in female hiPSC-CMs from each pathway, were analyzed using qRT-PCR, but these were not found to be statistically significant (**Figure S9**). To further interpret the gene expression data, a hallmark gene set analysis was conducted with female-derived hiPSC-CMs as a reference.

### Hallmark gene sets related to hypoxia were suppressed in male hiPSC-CMs

In male hiPSC-CMs, there is a notable suppression in the activity of the hallmarks of “Epithelial Mesenchymal Transition,” “Hypoxia,” “TNFA Signaling via NFKβ,” “P53 Pathway,” and “KRAS Signaling Up” gene sets relative to their female counterparts (**Figure 2J**). This reflects potential variations in stress response and common signaling pathways wherein female cells show some level of basal activation. When looking at heat maps of the hallmark genes that caused pathways enrichment by hiPSC line, it is apparent that these pathways tend to be more activated in female hiPSC-CMs than the male hiPSC-CMs (**Figure S10**). Additionally, multiple genes of interest can be identified in each heat map where most of the female hiPSC-CMs have higher expression than the male hiPSC-CMs. These genes have been highlighted in **Figure S10**. All of the information gleaned from this analysis reveals potential targets for non-hormone-mediated sex dimorphism in hiPSC-CMs. In total, this study reveals functional and genetic differences in hiPSC-CMs from males and females, making the inclusion of sex as a variable when studying hiPSC-CMs in vitro essential.

### Increased expression of SCN3B and sodium current in female hiPSC-CMs

**Figure 3.**
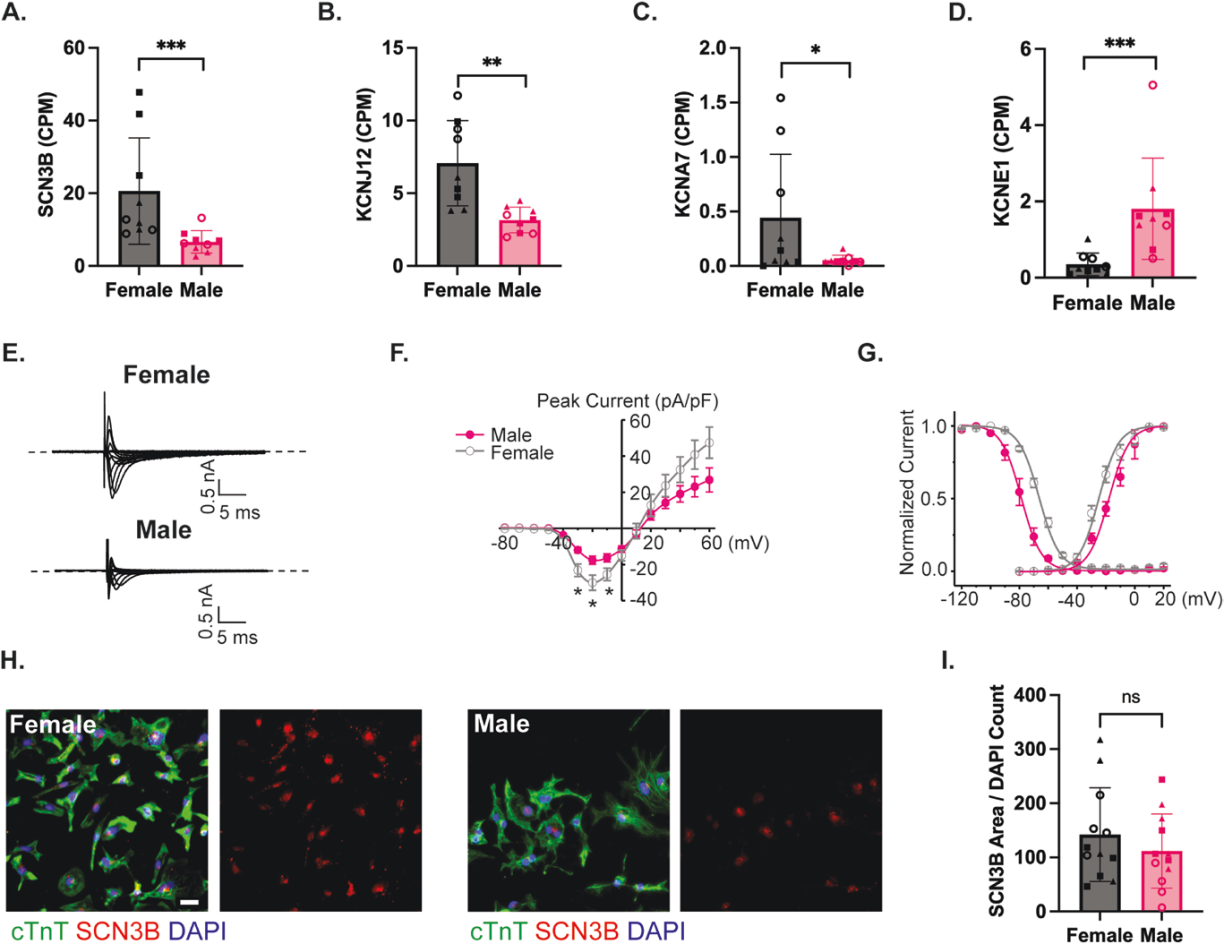
Differential ion channel-associated gene expression in male and female human induced pluripotent stem cell-derived cardiomyocytes (hiPSC-CMs). Graphs showing the expression level in counts per million (CPM) for the ion channel genes (A) sodium voltage-gated channel 3 B (*SCN3B*), (B) potassium inwardly rectifying channel subfamily J member 12 (KCNJ12), (C) potassium voltage-gated channel subfamily A member 7 (*KCNA7*), and (D) potassium voltage-gated channel subfamily E regulatory subunit (*KCNE1*). Sodium current recordings from hiPSC-CM patch-clamp are presented as (E) sodium current traces from male and female hiPSC-CMs, (F) current-voltage (I-V) curves, and (G) normalized activation and inactivation curves. (H) Immunocytochemistry for SCN3B (red), cardiac troponin T (cTnT: green) to mark cardiomyocytes, and DAPI (blue) to mark nuclei. Scale 50 μm. (I) Quantification of SCN3B area normalized to the DAPI count. For (A-D), the bar represents the average ± SD and each dot is the normalized expression level in CPM for one independent experiment with *n* = 3 replicates per lines and 3 hiPSC-CM lines per sex. Statistical significance designations are based on an Edge Test of raw reads and the false discovery rate corrected *P*-value. The test used to determine differentially expressed genes (DEGs). Where *P < .05, **P < .01, ***P < .001, and ****P < .0001. For (F-G), the data are presented as mean ± SEM. *n* = 10 for each group across 2 batches from 2 female and 2 male lines. A 2-sample Student’s *t*-test was used for statistical analysis with *P < .05. For (I), each data point represents the average of 5 fields of view across one well, and 2 wells were imaged per biologic replicate for all 6 lines. A 2-sample *t*-test was used for statistical analysis. For all bar graphs, ○: F5L1 and M4L1; ■: F6L1 and M7L1; and ▲: F7L1 and M9L1.

Due to differences in CaTs and previous reports of different ion channel and ion channel-associated genes in hiPSC-CMs, the list of DEGs was probed in a biased manner for any calcium-handling or ion channel genes (**Figure 3**). There were no calcium-handling genes identified, corroborating previous reports that the majority of calcium-handling differences are mediated by estrogen.^40,41^ However, we found significant differential expression of 4 ion channel-associated genes relevant to the cardiac action potential and pertinent to cardiac health and disease. Female hiPSC-CMs showed increased expression of (1) sodium voltage-gated channel beta subunit 3 (*SCN3B*), (2) potassium inwardly rectifying subfamily J member 12 (*KCNJ12*), and (3) potassium voltage-gated subfamily member 7 (*KCNA7*) (**Figure 3**A-C). Differential expression of these ion channel-associated genes in a healthy hormone-free system has, to our knowledge, never been reported. We also validate here an increase in potassium voltage-gated channel subfamily E regulatory subunit 1 (*KCNE1*) expression in male hiPSC-CMs reported in one previous study (**Figure 3D**).^28^ These results indicate baseline differences in ion channel-associated protein composition between male and female hiPSC-CMs and serve as a rationale for the inclusion of sex in pharmacological studies that target these channels and other channels that cooperate to generate the cardiac action potential. Additionally, qRT-PCR was performed on these ion channel-associated transcripts (**Figure S9**). *SCN3B* gene expression was found to be significantly upregulated in female hiPSC-CMs by qRT-PCR.

We decided to focus on SCN3B since increased expression has been linked to increases in sodium current in the myocardium.^42,43^ We used patch-clamp monitoring of sodium currents and found a significant increase in peak amplitude and sodium window current in the female hiPSC-CMs in comparison to male hiPSC-CMs (**Figure 2**E-G). Increases in peak current amplitude have been linked to increases in cardiac action potential upstroke velocity.^44^ In female hiPSC-CMs, I_NA_ activation curve shifted in the hyperpolarized direction and the inactivation curve shifted in the depolarization direction, resulting in a much larger window current than that in male hiPSC-CMs. An increase in window current is typically associated with a prolonged action potential duration (APD). This is particularly relevant with certain cardiac arrhythmias.^45^ To determine whether SCN3B expression was increased at the protein level, we used immunocytochemistry and quantified SCB3B area/DAPI count for multiple fields of view within multiple wells of hiPSC-CM (**Figure 2H**). Though not statistically significant, we found a trend towards increased SCN3B area/DAPI count in the female hiPSC-CMs (**Figure 2I**).

## Discussion

Here, we first established sex as an underreported and understudied variable in hiPSC-CM primary research literature. We then established a resource for studying sex differences in hiPSC-CMs by reprogramming 3 female and 3 male hiPSC lines from adult left ventricular adult fibroblasts. When differentiated into hiPSC-CMs, we discovered a slight but significant increase in the net calcium-handling ability of female hiPSC-CMs in comparison to male ones. Additionally, the female CMs had increased irregularity of spontaneous beat rate. We established that female and male hiPSC-CMs also had different patterns of gene expression with over 300 DEGs leading to distinct clustering. GO pathways analysis determined the most enriched pathways between the male and female hiPSC-CMs were those related to cell-cell adhesion and amine metabolism. Gene set enrichment determined significant suppression of pathways related to hypoxia, p53, KRAS, and TNFA signaling in the female hiPSC-CMs. Together, these results reveal baseline sex differences in hiPSC-CMs at the functional and transcript level without the addition of exogenous hormones.

Male and female hiPSC-CMs showed differential expression of multiple cardiac ion channel-associated genes that have implications for health and disease. First, we bolstered the findings of a previous research study that demonstrated increased expression of the KCNE1 gene in male hiPSC-CMs, making the female hiPSC-CMs more susceptible to proarrhythmias such as Torsades de Pointes.^28^ This study only looked at 2 male and 2 female lines, with only one replicate per line making these initial results tenuous with statistical significance difficult to establish. Our study confirms this finding across 3 male and 3 female lines with 3 independent hiPSC-CM differentiation batches for every line. The primary function of KCNE1 in CMs is regulating the cardiac APD.^46^ It serves as a major contributor to the QT interval, or the repolarization current of CMs.^46^ As mentioned above, KCNE1 is implicated in genetic and drug long-QT syndrome and Torsades de Pointes which are arrhythmias more common in women.^47,48^ These results confirm sex is an important variable to consider in pharmacological studies on hiPSC-CMs, especially for drugs that act as potassium channel blockers.

We also discovered that females have significantly higher expression of the sodium ion channel SCN3B, and the potassium channels KCNJ12 and KCNA7. SCN3B is a sodium ion channel auxiliary subunit and increases in SCN3B gene expression have been correlated to increases in sodium current in myocardial cells.^42^ Conversely, knockouts and mutations of the SCN3B gene in mice show decreased cardiac sodium current density and abnormal electrophysiology.^43,49^ We further looked at sodium current in male and female hiPSC-CMs and found increases in the peak amplitude and window current of female hiPSC-CMs. Mutations in SCN3B have also been linked to the onset of atrial fibrillation, an arrhythmia more prevalent in men.^6,49^ The lower basal expression of SCN3B in non-diseased male CMs could be a mediator of this disparity which provides the scientific premise for future research. Another key ion channel-associated gene, we found to be present at higher levels in female hiPSC-CM, was KCNJ12. Expression of this gene is decreased in hearts with dilated cardiomyopathy in comparison to healthy controls, making decreases in KCNJ12 expression either a consequence of the disease or a potential mediator of it.^50^ Interestingly, nongenetic dilated cardiomyopathy is a more common phenotype seen in men with HF.^5^ Taken together, these results indicate that sex differences in ion channel expression in cardiac health could be mediators of sex differences in cardiac disease.

Our investigation also highlights hiPSC-CM baseline sex differences in calcium handling, somewhat corroborating existing findings regarding sex differences in human and animal hearts. One study showed that female rat hearts expressed significantly more ryanodine calcium release channel (RyR), the L-type calcium channel IC (CANCA1 or Cav1.2), and the sodium-calcium exchange channel (NCX).^51^ Later studies attributed these differences to sex hormones. A study looking at rabbit hearts saw an increase in calcium handling spurred by increases in L-type calcium channels and sodium-calcium exchanges in the apex of female rabbit hearts.^40^ When the cells were excised, the exogenous addition of estrogen led to a doubling in the calcium current in CM from the rabbit heart apex. This was later validated in primary human ventricle tissue where female apex tissue had a higher expression of L-type voltage-dependent channel Cav1.2 and the sodium-calcium exchanger NCX1.^41^ This result was seen only in premenopausal women, indicating estrogen might play a role. When male and female hiPSC-CMs were conditioned with estrogen, both the sodium and calcium current increased in the female CMs but not in the male CMs.^41^ Our findings confirm that hormones are likely the cause for the differential expression of Cav1.2, RYR, and NCX as none of those genes were differentially expressed in our dataset. However, our findings also suggest that female CMs have a slight but significant increase in calcium handling even without the addition of exogenous hormones. The etiology for these changes could be due to the increases in sodium current peak amplitude seen in the female hiPSC-CMs that we suggest is mediated in part by increased SCN3B expression. Rapid depolarization of CMs caused by sodium influx is what sparks the sodium-calcium exchange in CM excitation-contraction coupling.^52^

Notably, we also observed significant differences in cell-cell adhesion pathways via bulk RNA sequencing. Cell adhesion processes are not just mechanical connections between cells but are also crucial for the transmission of signals between cells. These are especially crucial in highly mechanically active organs such as the heart.^53^ In our data set, DEGs in these pathways revealed differences in female and male CDH expression. The expression levels of CDH1 and CDH17 were upregulated in female hiPSC-CMs, while CDH7 and CDH12 were upregulated in male hiPSC-CMs. Although N-cadherin (CDH2) is more abundant in the heart and plays a critical role in intercalated disk assembly, CDH1 can also be found in the heart.^54,55^ In a genetic rat model of cardiac hypertrophy, the hypertrophic rats showed significantly higher levels of CDH1 compared to healthy rats, and this expression was particularly concentrated at the CM intercalated discs.^55^ This suggests that altered cell adhesion, through the expressions of cadherins, may play a role in cardiac hypertrophy. The basal differences in cadherin expression between male and female hiPSC-CMs could be mediators of sex dimorphism in cardiac hypertrophy and merit future study.^56^ Future research will also be needed to determine whether gene expression changes related to cell-cell adhesion are observed at the protein level and affect functional cell adhesion.

DEG from the Bulk RNA Sequencing Analysis was used to create a Volcano Plot, visually representing the gene expression variations between CMs derived from males and females. As expected, some of the DEGs were located on the Y chromosome, which is exclusive to males. The role of Y-linked genes in cardiac health and disease is significant. For instance, an expression profiling study of patients with new-onset HF due to idiopathic dilated cardiomyopathy identified a marked upregulation in USP9Y, DDX3Y, RPS4Y1, and EIF1AY, correlating with pronounced fold changes in male patients.^57^ Additionally, research comparing control subjects to HF patients found that out of 7 highly expressed gene IDs in the HF group, 5 were Y-linked genes: EIF1AY, RPS4Y1, USP9Y, KDM5D, and DDX3Y.^58^ All of these Y-linked genes were significantly increased in the male hiPSC-CMs of this study. The precise contributions of these Y-linked genes to cardiac health and disease remain underexplored, presenting another potential avenue for future research.

The separate bulk RNA sequencing clustering of the male and female hiPSC-CMs is significant as other studies have determined that, even in large cohort studies encompassing hiPSCs from 40 donors, hiPSCs themselves cluster independently of sex and age.^59^ This is significant to note as it shows that the inherent cardiac sex dimorphism of hiPSC-CMs is great enough to be detected even with a cohort of 6 lines. Though some of the most significantly DEGs are Y-linked, the sex differences are not necessarily seen in all cell types but are unique in this case to the hiPSC-CMs themselves.

Of note, the hiPSC-CMs of this study exhibit maturity levels of the fetal period.^60^ Future studies could impose maturation conditions on the hiPSC-CMs, such as metabolic maturation media, electrical stimulation, or 3D engineered heart tissue culture.^61-65^ A recent review eloquently laid the framework for the future of studying sex in more complex engineered tissues, often including multiple cell types.^66^ In addition to studying the phenotype after maturation conditions are imposed, mechanistic information can be gleaned as to how female and male CMs mature. This is highly relevant as there is sex dysmorphism in the presence and frequency of various congenital heart defects.^67^ Additionally, a recent study looking at single-cell RNA sequencing data at different developmental time points showed that CMs of different sexes had the most DEGs at multiple developmental times. This was largely mediated by sex-specific progesterone receptor differences driving maturation.^68^ Another future consideration would be to add hormones. By not adding hormones, we were able to see baseline sex differences in the hiPSC-CMs that are not driven by hormones. Future studies might include the addition of hormones since, as described above, estrogen plays a key role in cardiac development, health, and disease.^69,70^

A challenge of working with human source material is line-to-line variability which limits our ability to detect subtle differences between sexes. One approach to address this issue is to increase the number of male and female lines. There was also a recent study that generated isogenic hiPSC lines with various sex chromosomes: XY, XX, XXY, and X0. In the future, banks of lines such as these could be used to study sex differences without differences in non-sex chromosome genetic background.^71^ This also brings up the fact that our study only looked at the dominant sex chromosome orientations, XX and XY, where there could be other basal differences in individuals with Klinefelter syndrome (XXY), Turner syndrome (X0), or other sex chromosome abnormalities. These other sex chromosome variants should be considered in future studies.

Along the lines of sex chromosomes, the hiPSC somatic cell reprogramming process is limited in itself because of the high tendency for hiPSCs to contain irregularities in X chromosome inactivation.^72,73^ This is limiting when studying sex differences using hiPSC-derived cells as genes that escape X-inactivation can be mechanistic mediators of cell-level sex dimorphism.^74,75^ Two of the female lines used in this study had a notable decrease in XIST, a gene that regulates X-chromosome inactivation (**Figure S6**). Thus, the 14 DEGs that are linked to the X chromosomes in our study might be atypical for female CMs. However, we see that the differential expression of X-linked DEGs was not significantly influenced by the erosion of XIST expression in F5 and F6 lines. In addition, only a very small fraction (4%) of DEGs identified in this study was located on the X chromosome. Of these genes, only one is involved in cell-cell adhesion (IGSF1). The others (eg, PLS3, CTPS2, ZRSR2, KLHL4, SYAP1, ATT2, RTL9, VCS3B) are not associated with the primary processes we identified as sex dimorphic in hiPSC-CMs (eg, amine metabolism, hypoxia, sodium current dynamics). Finally, the vast majority of DEGs were autosomal or Y-linked, suggesting that XCI erosion did not significantly affect the observed sex differences in gene expression and CM function in this study. Even so, last year it was discovered that reprogramming hiPSCs through a naive state highly reduced abnormalities in hiPSC X-inactivation along with other epigenetic abnormalities acquired with reprogramming.^76^ In the future, adoption of this, or similar reprogramming methods, could be used when studying sex differences using hiPSCs.

Overall, our study demonstrates that sex-related differences are present in hiPSC-CMs even without the addition of exogenous hormones. This was achieved using adult left ventricular fibroblasts with the most common SeV reprogramming technology, in an attempt to limit epigenetic differences in cell sourcing and reprogramming. These findings highlight the importance of considering sex as a variable in hiPSC-CMs and identify novel mediators of cardiac sex dimorphism that are not mediated by exogenous sex hormones. So that these outcomes and others related to CVD can be more easily studied, we provide an essential resource for the field in the form of a controlled cohort of male and female hiPSCs.

## Supplementary Material

sxaf038_suppl_Supplementary_Figures_S1-S10_Tables_S1-S2

## Data Availability

All data are incorporated into the article and its online Supplementary Material. The raw bulk RNA sequencing data are available on GEO at GSE293391.
