# Supplementary material for: Healthy human induced pluripotent stem cell-derived cardiomyocytes exhibit sex dimorphism even without the addition of hormones
Source: Stem Cells. 2025 Jun 20;43(9):sxaf038. doi: 10.1093/stmcls/sxaf038 (PMC12371235; doi:10.1093/stmcls/sxaf038)
Supplement: sxaf038_suppl_Supplementary_Figures_S1-S10_Tables_S1-S2 [file sxaf038_suppl_supplementary_figures_s1-s10_tables_s1-s2.pdf]

Supplementary Information: Healthy human induced pluripotent stem cell-derived cardiomyocytes exhibit sex dimorphism even without the addition of hormones

Sophie E. Givens[1]*, Abygail A. Andebrhan[1]*, Eric G. Schmuck[2], Aimee Renaud[3], An Xie[4], Somayeh Ebrahimi-Barough[1], Juan E. Abrahante[5], Noah Stanis[1], Samuel Dudley[4], James R. Dutton[3,7,8], Brenda M. Ogle[1,3,6,7]

[1]Biomedical Engineering, University of Minnesota, Minneapolis, MN, USA
[2]Stem Cell & Regenerative Medicine Center, University of Wisconsin-Madison. Madison, WI, USA
[3]Stem Cell Institute, University of Minnesota, Minneapolis, MN, USA
[4]Lillehei Heart Institute, University of Minnesota, Minneapolis, MN, USA
[5]Informatics Institute, University of Minnesota, Minneapolis, MN, USA
[6]Department of Pediatrics, University of Minnesota, Minneapolis, MN, USA
[7]Institute of Engineering in Medicine, University of Minnesota, MN, USA
[8]Department of Genetics, Cell Biology and Development, University of Minnesota, Minneapolis, MN, USA
* Indicates first authorship

Supplemental Materials and Methods

Literature Search

The goal of this literature search was to analyze publications from PubMed between 2010 and 2023 to assess whether sex as a biological variable has been considered in hiPCS-CM research. We began broadly, using PubMed to identify papers on hiPSC-CM with the search term "(Human Induced Pluripotent Stem Cell derived Cardiomyocytes) OR hiPSC-CM OR (human iPSC-CMs)". We tracked the annual count of such papers from 2010 to 2023. Next, we refined our search to include "(sex OR male OR female)" to determine whether any of these terms were mentioned in the context of hiPSC-CMs. To hone in even further, we refined our search to

include "(sex AND male AND female)", to determine if any hiPSC-CM publication contained all of these terms. Recognizing the limitations of this approach, we conducted a more thorough search. We input "(Human Induced Pluripotent Stem Cell derived Cardiomyocytes) OR hiPSC-CMs OR (human iPSC-CMs)" into PubMed and reviewed the first 50 primary research articles listed for each year from 2010 through 2023. In each publication reviewed we determined whether the sex of the hiPSC-CM cell lines was reported and then whether the results were analyzed by sex.

Adult left ventricular cardiac fibroblasts (aLVCF) isolation

All human tissue procurement and use was performed with local Institutional Review Board approval. Donor human hearts that were considered healthy based on available clinical information, but still went unmatched for transplant were obtained from the University of Wisconsin Organ Procurement Organization, Madison, WI. Donor hearts associated with underlying coronary artery disease, impaired left ventricular function (ejection fraction <50%), known bacteremia, viral hepatitis or HIV, or anticipated cold ischemic time of >6 hours were excluded. The aLVCF isolation was performed as previously described[83–86]. Briefly, hearts were flushed with ice-cold cardioplegia solution, excised and immediately submerged in ice-cold cardioplegia solution, and transported on ice. 25-30 g of left ventricle was excised, minced, distributed equally into 4 gentleMACS C-tubes, and homogenized with a gentleMACS tissue dissociator (Miltenyi Biotec, Bergisch Gladbach, Germany). 10 ml of digestion media containing DMEM (Corning, Corning, NY) and 2.5 mg Liberase TM (Roche, Basel, Switzerland) were added to each tube and incubated at 37 °C for 30 min with constant agitation. Heart samples were sieved through a 200 µm filter and then centrifuged at 1000×g for 20 min. The cell pellet was suspended in 20 ml complete media (MCDB 131 with 10% FBS (R&D Systems, Lot E20074), 1 ng/ml basic fibroblast growth factor (bFGF), 5 µg/ml insulin, 10 µg/ml ciprofloxacin and 2.5 mg/ml amphotericin B) and plated into two T75 flasks. Cells were permitted to attach for 2 hours, which is a time that offered an optimal CF yield with high purity during earlier protocol optimization studies. Non-adherent cells were removed by washing in 1X phosphate-buffered saline (PBS). Finally, complete media was added, and the cells were cultured at 37 °C, 5% CO_2, and 100% humidity, and passaged once they were ~90% confluent.

LVCF reprograming into human-induced Pluripotent Stem Cells (hiPSCs)

The aLVCF lines were obtained via MTA (MSN251847) using an IRB-governed protocol at the University of Wisconsin-Madison. For this study, six hiPSC lines were used, all originating from left ventricular cardiac fibroblasts. Three lines were from female donors (F7, F6, and F5-LVCFs) and three lines from male donors (M4, M7, and M9-LVCF) between the ages 21 and 34 with no cardiac abnormalities (**Table S1**). The LVCFs were reprogrammed using the Cyto-tune 2.0 (Thermo A16517) Sendai Reprogramming Kit according to the suppliers' directions. Briefly, low passage LVCFs, between passages 1 and 3, were plated at a density of ~7000 cell/cm^2 in LVCF-media (MCDB131 Low Glucose, 10% FBS, 5 μg/mL recombinant human insulin and 1 ng/mL recombinant human basic fibroblast growth factor). The following day Cyto-tune 2.0 Sendai virus was added to the LVCF-media and removed 24-hours later. The cells were maintained in LVCF-media for 6 more days. On day 7, the cells were singularized using Accutase and replated at a 1:6 ratio onto a vitronectin (Thermo A31804) coated plate. To coat plates with vitronectin, the vitronectin was diluted to a 10μg/mL concertation in DPBS minus calcium and magnesium and left at 4°C overnight or at least 1-hour at room temperature. The day following replating, the media was changed to TesR-E8 (STEMCELL 05990), and the cells were maintained in TesR-E8 for the next 2-3 weeks while colonies began to emerge. Independent colonies that looked pluripotent were manually passaged using a microscope and Pasteur pipette tip in the biosafety hood. Approximately 12-18 colonies were selected for each line and each colony was manually passaged 5-times to aid exogenous Sendai vector elimination. After 5-manual passages colonies that were maintaining a pluripotent colony phenotype were selected and bulk passaged using Versene (Thermo A423101) onto vitronectin-coated plates. Between passage 8-20 6 colonies were tested via q-RT-PCR for Sendai vector using the primer pair recommended in the Cyto-tune manual (**Table S4**). A line was considered Sendai vector depleted when no SEV product was detected for two passages via qPCR for 40-cycles. Three clones that were Sendai vector depleted were expanded and banked using mFresR (STEMCELL 05855). One Sendai vector-depleted clone from each line was chosen to move forward with for comprehensive pluripotency assessment, karyotyping, and cardiomyocyte differentiations (F7C6, F6C2, F5C9, M4C8, M9C10, and M7C16-hiPSCs). This assessment took place after Sendai

virus depletion was confirmed and a master cell bank of each line was created using mFresR (STEMCELL 05855). For ease of use the lines later remained as the donor identifier (e.g. F5) followed by L1, L2, or L3 for lines 1, 2, and 3 as shown in **Table S1**.

Pluripotency assessment

The pluripotent status of the hiPSC-lines was assessed via q-RT-PCR, flow cytometry, and immunocytochemistry (ICC). For q-RT-PCR the cells were lysed, and RNA was purified using the PureLink™ RNA Mini Kit (Thermo 12183025). The purified RNA was converted to cDNA using SuperScript™ IV VILO™ Master Mix. The qPCR was completed using forward and reverse primers for OCT4, SOX2, and NANOG with SYBR Green PCR Master Mix (Thermo 4367659). The primer sets are shown in **Table S4**. The ΔCt was calculated in comparison to the reference gene GAPDH and was compared to aLVCF as a negative control and a previously characterized hiPSC-line, CCND2-hiPSCs as a positive control[42]. To confirm the presence of an undifferentiated state at a protein level, flow cytometry for OCT4 and SSEA4 was conducted with the detailed methods outlined below. Additionally, four markers that indicate an undifferentiated state were stained for, OCT4, SSEA4, SOX2, and TRA-1-60, using ICC following the suppliers' directions from the Pluripotent Stem Cell 4-Marker ICC Kit (**Table S3**). This assessment was done within 5 passages of creating a master cell bank.

hiPSC-lines Karyotyping

Adherent cells were harvested with colcemid arrest (Irvine Scientific), treated with 0.75 M KCl hypotonic solution, and fixed with 3:1 methanol:acetic acid. The resulting cells were spread onto glass slides according to standard cytogenetic protocols, aged in a 90-degree oven for 1.5 hrs and stained with Wright's/Giemsa stain. Twenty G-banded metaphases were evaluated using an Olympus BX51 microscope outfitted with 10x and 100x objectives and karyotyped using Applied Spectral software. Olympus BX51 with 60x and 100X oil objective lenses. Interferometer-based CCD cooled camera. Software for G-bands and SKY: Applied Spectral Imaging (ASI) BandView. This assessment was done within 5 passages of creating a master cell bank.

Cell maintenance of hiPSCs

The hiPSCs were cultured in mTesR™1 (STEMCELL 85850) on 8.68 ug/cm^2 (or 0.5 mg per 6 well-plate) Cultrex reduced growth factor (RGF) (R&D Systems 3433-005-01) coated 6-well plates. The media was changed into 2mL of fresh mTesR every day. Upon reaching 70-80% confluency, hiPSCs were passaged using ReLesSR™ (STEMCELL 05872). The exhausted media was aspirated, and the hiPSCs were washed with 1 mL of DPBS (-) calcium (-) magnesium to remove residual medium and contaminants. Then, 1 mL of room temperature ReLesSR™ was added to each well, followed by a 45-second incubation. After aspirating the ReLesSR™, the plate was placed in a 37°C incubator for 7 minutes. After the incubation, 1 mL of warm mTeSR™1 was added to each well, and the hiPSC colonies were detached from the bottom by gently tapping the sides of the plate. The cell suspension was diluted at a 1:6 ratio and transferred into a new Cultrex-RGF coated 6-well plate at 2mL cell suspension per well. The stem cells used for experimentation were used within 10 passages of the creation of a master cell bank. The cell lines were not authenticated. One female (F6L1) and one male line (M4L1) were shown to be negative for mycoplasma via ATCC authentication services. The sterility of the cells for bacterial and fungal contaminants was observed daily via brightfield microscopy.

Differentiation and purification of hiPSC-derived Cardiomyocytes (hiPSC-CMs)

Upon reaching 70-80% confluency, hiPSCs were dissociated using Accutase solution (Millipore Sigma A6964). The exhausted media was aspirated, and cells were washed with 1 mL of DPBS (-) calcium (-) magnesium. Then, 1 mL of room temperature Accutase solution was added to each well and incubated at 37°C for 8 minutes. The hiPSCs were then singularized by pipetting up and down with a P1000 micropipette and quenched using ½ mL of mTesR. The resulting cell suspension was transferred to a conical tube and counted on a hemocytometer using a 1:1 dilution of 0.4% trypan blue. The hiPSCs were then seeded onto a 12-well plate coated with 8.68 ug/cm^2 Cultrex-RGF at a density of 0.5x10^6 cells per well in 1 mL of mTesR with 5uM Rock Inhibitor. The exhausted media was replaced every day with 2 mL of fresh mTesR until the cells reached 97-100% confluency (2-4 days). Upon reaching confluency (defined as day 0 of

differentiation), the media was replaced with RPMI/B27(-insulin) (Fisher A1895601) containing varied CHIR99021 (Sigma SML1046) concentrations based on the hiPSC line (**Table S1**). On day 2, exactly 48 hours later, the media was replaced with RPMI/B27(-ins) containing 7.5 uM of IWP2 (Tocris 686770-61-6), followed by another media change with RPMI/B27(-ins) on day 4. The media was then replaced every two days until day 13 of differentiation, using fresh RPMI/B27(+ins) (Thermo 17504001).

On day 13, the cardiomyocytes were replated onto 8.68 g/cm^2 Cultrex-RGF coated 12-well plates at a 1:2 ratio to facilitate lactate purification. Each well was washed with 1/2 mL of DPBS (-) calcium (-) magnesium. Subsequently, 1 mL of 37°C 0.25% trypsin-EDTA solution was added to each well and incubated at 37°C for 20 minutes. After incubation, cardiomyocytes were singularized by pipetting with a P1000 micropipette, then quenched with 2 mL of RPMI/B27(+ins) with 20% FBS. The resulting cell suspension was transferred to a conical tube, centrifuged at 200g for 5 minutes, and the supernatant was aspirated. Cells were resuspended in RPMI/B27(+ins) containing 10 uM Rock Inhibitor and seeded at 1mL cell suspension per well. On day 14, the replated cells were given 1 mL of RPMI/B27(+ins). Cardiomyocytes were then purified using lactate purification media composed of DMEM no glucose with 4 mM lactate. On day 15, the media was replaced with 1 mL lactate medium. On day 17, the cells were washed with 1/2 mL of DPBS (-) calcium (-) magnesium, and the media was replaced with 1 mL of lactate medium. On day 19, the cells were washed with 1/2 mL of DPBS (-) calcium (-) magnesium and switched into RPMI/B27 (+ins). The media was changed every three days with fresh RPMI/B27(+ins) until the cardiomyocytes were lifted for flow cytometry and RNA lysis on day 40, or for Calcium Imaging on day 60.

Flow Cytometry

Flow cytometry was used to assess hiPSCs pluripotency and hiPSC-derived cardiomyocyte purity after lactate purification. For cell fixation and permeabilization, the hiPSCs were singularized using Accutase (the same method as used above). After incubation, the cells were quenched with 1/2 mL of mTeSR per well, and the resulting cell suspension was centrifuged for 5 minutes at 200g. The supernatant was removed, and the cells were fixed in 1%

paraformaldehyde for 20 minutes and centrifuged again for 5 minutes at 200g. The PFA was removed, and the cells were resuspended in 90% v/v methanol and stored at -20°C for up to 6 weeks. For cardiomyocytes, on day 40, each well was washed with 1/2 mL of DPBS (-) calcium (-) magnesium and incubated with 1/2 mL of 37°C 0.25% trypsin-EDTA for 20 minutes at 37°C. After incubation, the cardiomyocytes were singularized with a P1000 micropipette and quenched with 1 mL of RPMI/B27(+ins) with 20% FBS. The cells were transferred into a conical tube and centrifuged for 5 minutes at 200g. The supernatant was removed, and the cardiomyocytes were fixed in 1% paraformaldehyde (PFA) for 20 minutes and centrifuged for 5 minutes at 200g. The PFA was removed, and the cells were then resuspended and stored in 90% v/v methanol at -20°C for up to 6 weeks. For flow cytometry stains the fixed cells were washed out of the methanol with 2 mL of flow buffer (PBS with 0.1% Triton-X and 0.5% w/v BSA) twice. The cell pellet was then resuspended in 100uL of primary antibody at the dilution indicated in **Table S3**. The fixed cells were incubated in primary antibody for 1 hour at room temperature and then washed twice with 2 mL of flow buffer. If the primary antibody was conjugated to a fluorophore the control was an isotype conjugated to the same fluorophore and the cells were resuspended in 300uL of flow buffer and 10,000 events were acquired on a BD Accuri C6 Flow Analyzer. For stains with unconjugated primary antibodies, the cell pellet was resuspended in 100uL of secondary antibody and incubated at room temperature for 30 minutes. The fixed cells were washed twice with 2mL of flow buffer, resuspended in 300uL of flow buffer, and then 10,000 events were acquired on a BD Accuri C6 Flow Analyzer. The negative control for the cardiac troponin T stains were undifferentiated hiPSCs.

Calcium transients of Cardiomyocytes

On day 60, cardiomyocytes from each cell line were incubated with 5uM Rhod-2AM calcium-sensitive dye in LASR medium for 30 minutes at 37°C. Following the incubation period, the dye was removed and replaced with Tyrode's Salt Solution containing 1 g/L sodium bicarbonate (TSS, Sigma Aldrich T2145). The cells were allowed to equilibrate in TSS for 30 minutes at 37°C and were subsequently subjected to imaging on the TxRed channel using a 30ms exposure and a gain of 1.0 at 10X magnification on a fluorescent microscope (Leica Microsystems). For each well, three spontaneous and three-paced 20-second videos were captured, with n=3 wells

per condition for each replicate. For the videos taken with electrical stimulation, the cells were paced using a platinum electrode at 1 Hz with a voltage of 7 V and a pulse duration of 0.02 ms. The calcium transient videos acquired were processed using ImageJ. Three regions of interest within each video were selected, and their respective Z-axis profile was saved as .txt files. The text files were analyzed using a MATLAB code to determine the inter-spike interval, time to peak, maximum amplitude, as well as the average upstroke and downstroke velocities.

Bulk RNA Sequencing

On day 40, cardiomyocytes were lifted in the same manner as for flow (described above). The cell pellet was quickly resuspended in a solution of 700µL PureLink Lysis buffer containing 7µL Beta-mercaptoethanol. The cell lysis was quickly transferred to -80°C for storage. The RNA purification was done using the PureLink^{TM} RNA Mini Kit (Thermo 12183025). The DNA was removed using the on-column Ambion PureLink DNase Set (Thermo 12185010). Total eukaryotic RNA isolates are quantified using a fluorimetric RiboGreen assay. Total RNA integrity is assessed using capillary electrophoresis (e.g. Agilent BioAnalyzer 2100), generating an RNA Integrity Number (RIN). For samples to pass the initial QC step, they need to quantify higher than 500 ng and have a RIN of 8 or greater. Total RNA samples are then converted to Illumina sequencing libraries. Total RNA samples are converted to Illumina sequencing libraries using Illumina's TruSeq RNA Sample Preparation Kit (Cat. # RS-122-2001 or RS-122-2002) or Stranded mRNA Sample Preparation Kit (Cat. # RS-122-2101). (Please see www.illumina.com for a detailed list of kit contents and methods). In summary, the mRNA from a normalized input mass of total RNA is isolated using oligo-dT coated magnetic beads, fragmented, and then reverse transcribed into cDNA. The cDNA is blunt-ended, A-tailed, and indexed by ligating molecularly barcoded adaptors. Libraries are amplified using 15 cycles of PCR. The final library size distribution is validated using capillary electrophoresis and quantified using fluorimetry (PicoGreen) and via Q-PCR. Indexed libraries are then normalized, pooled, and size-selected to 320 bp (tight) using the PippinHT instrument. Pooled libraries are denatured and diluted to the appropriate clustering concentration. The libraries are then loaded onto the NovaSeq paired end flow cell and clustering occurs on board the instrument. Once clustering is complete, the sequencing reaction immediately begins using Illumina's 2-color SBS chemistry. Upon

completion of read 1, a 7 base pair index read is performed in the case of single indexed libraries. If dual indexing was used during library preparation, 2 separate 8 or 10-base pair index reads are performed. Finally, the clustered library fragments are re-synthesized in the reverse direction thus producing the template for paired end read 2.

Bulk RNA Sequencing Analysis

2 x 50bp FastQ paired-end reads for 6 samples (n=62.4 Million average per sample) were trimmed using Trimmomatic (v 0.33) enabled with the optional "-q" option; 3bp sliding-window trimming from 3' end requiring minimum Q30. Quality control on raw sequence data for each sample were performed with FastQC. Read mapping was performed via Hisat2 (v2.1.0) using the Human genome (GRCh38) as a reference. Gene quantification was done via Feature Counts for raw read counts. Differentially expressed genes were identified using the edgeR (negative binomial) feature in CLCGWB (Qiagen, Redwood City, CA) using raw read counts. We filtered the generated list based on a minimum 2x Absolute Fold Change and FDR corrected $p < 0.05$. Differentially Expressed Genes (DEGs) were sorted based on log2(fold change) and -log10(false discovery rate, FDR). These values were then visualized in a Volcano Plot using the VolcaNoseR tool. Known Y-linked genes were filtered using the National Center for Biotechnology Information's Gene database. Over-representation Analysis (ORA) and Gene Set Enrichment Analysis (GSEA) were performed on the differential gene expression results table(s) using the ClusterProfiler_wrapper v2.1 module at the Minnesota Supercomputer Institute. This script is a wrapper for the clusterProfiler R package v4.7.1.003 for R v4.2.2[87,88]. Over-representation analysis was performed on 275 DEGs genes after applying an FDR cutoff of 0.05, a log2(fold change) cutoff of 1, and a top number of genes cutoff of 400. The gene SYMBOLs were converted into ENTREZ IDs using the bitr() function for compatibility with the enrichGO (ontology=biological process, pvalueCutoff =0.05, qvalueCutoff = 0.2) and enrichKEGG (pvalueCutoff =0.05, qvalueCutoff = 0.2) clusterProfiler functions, and the enrichPathway (pvalueCutoff =0.05, qvalueCutoff =0.2) ReactomePA R package function[89]. The human genome-wide annotation database, 'org.Hs.eg.db' v3.16.0 R package, was used for gene conversions and the enrichment functions. The universe or background gene list that was provided to the enrichment functions included all tested genes in the differential gene expression

results table. All of the tested genes were ranked by their log2 fold change values, and GSEA was performed on the ranked list using hallmark database pathways (qvalue_threshold = 0.05) from the msigdbr R v7.5.1 package[90]. Genes permutations were performed for each pathway 10,000 times to calculate the normalized enrichment score (NES). Dotplots were generated with the ggplot2 R package.

Patch Clamp

hiPSC-CMs were trypsinized (0.25% trypsin-EDTA, Invitrogen) for 20 min and plated in 35 mm culture dishes at a cell density of ~5,000 cells/cm^2 on the day before the experiments. Na+ channel currents were measured by using the whole-cell patch-clamp technique in the voltage-clamp configuration at room temperature. hiPSC-CMs were not selected by action potential morphology, but the differentiation technique used results in predominantly ventricular-like cells. To measure Na+ channel currents, pipettes (~2 MΩ) were filled with a pipette solution containing (in mmol/L): CsCl 80, cesium aspartate 80, Aspartatic acid 80, EGTA 11, MgCl2 1, CaCl2 1, HEPES 10, and Na2ATP 5 (adjusted to pH 7.4 with CsOH). The bath solution consisted of (in mmol/L): NaCl 5, NMDG 105, TEA-Cl 20, CsCl 5, CaCl2 2, MgCl2 1.2, HEPES 10, 0.01 Nifidipine and glucose 5 (adjusted to pH 7.4 with CsOH). For activation, the holding potential was -100 mV. A voltage step protocol ranging from - 80 to +60 mV with steps of 10 mV was applied to establish the presence of Na+ channel activation currents. For inactivation, A voltage step protocol ranging from -120 to +30 mV with steps of 10 mV (400ms) followed by -20 mV (40ms) was applied. The holding potential was -120 mV for inactivation. The peak current density was used to plot current-voltage (I-V) curves. As before, nifedipine (10 µM, Sigma) was added in the bath solution to block L-type Ca2+ channel currents. Steady state activation and inactivation were characterized by Boltzmann functions.

Immunocytochemistry of SCN3B

At the endpoint of co-cultures, day 40, the 2D hiPSC-CM monolayers were singularized using 0.25% Trypsin-EDTA for 20 minutes at 37°C and replated onto 17.36 µg/mL Cultrex-RGF coated 48-well plates at a density of ~10,000 cells/cm^2. After 24 hours the cells were fixed in

diastole using 4% paraformaldehyde in PBS with 90mM KCl, 4mM EDTA, and 4mM $MgCl_2$ for diastolic arrest of the CM. The chamber slides were stored in PBS with 0.1% sodium azide at 4°C until staining. To stain, samples were permeabilized using 0.2% Triton-X-100 for 1 hour, blocked in BGST (50g/L BSA, 10g/L glycine, 2% Goat Serum, 0.1% Triton-X-100) for 2 hours and then incubated with a 1:200 dilution of anti-SCN3B antibody (Abcam Cat#: ab254861) and anti-cardiac troponin 2 (cTnT) at a 1:200 dilution (Fisher Cat#: MS295P1) for 3 hours at room temperature. The primary antibody was washed off and the cells were incubated with AlexaFluor-488 and AlexaFluor-647 Goat anti-mouse Goat anti-mouse secondary antibodies for 1 hour. The samples were incubated with 1g/L DAPI in PBS for 20 minutes and washed with PBS 3 times. The 2D wells were imaged at 20X magnification on a fluorescent microscope (Leica).

q-RT-PCR Validation

The RNA was isolated and converted to cDNA as described in the Bulk RNA Sequencing methods section. Gene expression analysis was performed using q-RT-PCR with SYBR Green chemistry (Thermo Fisher Scientific cat# 4367659). Each reaction was set up in a 15 µL final volume containing 7.5 µL of SYBR Green Master Mix, 0.75 µL of forward and reverse primers (100 µM), 1 µL of diluted cDNA (1:10), and 5.75 µL of nuclease-free water. All reactions were performed with triplicate technical replication. Gene expression levels were normalized to housekeeping genes (EDF-1), and presented as $2^{-\Delta Ct}$. Two hiPSC-CM batches for each line were assessed using q-RT-PCR.

Statistics

Statistics for bulk RNA sequencing analysis are described in the bulk RNA sequencing method sections. Comparisons of merged female and male functional data were done using a student t-test. Other statistics were conducted using a one-way ANOVA and post-hoc Tukey HSD comparison of means.

Table S1. Adult left ventricular fibroblasts donor demographic information.

Line	Sex	Age	Race	Heart Disease Assessment	Genetic Testing
F5	F	26	White	Negative history, normal EF by echocardiogram	None
F6	F	34	White	Negative history, normal EF by echocardiogram	None
F7	F	33	White	Negative history, normal EF by echocardiogram	None
M4	M	21	White	Negative history, normal EF by echocardiogram	None
M7	M	21	White	Negative history, normal EF by echocardiogram	None
M9	M	23	White	Negative history, normal EF by echocardiogram	None

Table S2. Donor line naming, SeV depletion and hiPSC-CM differentiation information.

Donor Line	Sex	Age	Line Name	Clone Name	Passage of SeV Depletion	Karyotype	Days to 70-90% Conf. (VTN in E8)	Days to 70-90% Conf. (MG in mTesR)	hiPSC-CM Dif. Seeding Density	hiPSC-CM Dif. CHIR (μM)
**F5**	**F**	**26**	**F5L1**	**F5C9**	**P18**	**XX (QC)**	**4-5**	**4-5**	**132K/cm²**	**7.5**
			F5L2	F5L5	P15					
			F5L3	F5L6	P14					
**F6**	**F**	**34**	**F6L1**	**F6C2**	**P16**	**XX (QC)**	**5-6**	**4-5**	**197K/cm²**	**6.5**
			F6L2	F6C5	P19					
			F6L3	F6C10	P14					
**F7**	**F**	**33**	**F7L1**	**F7C6**	**P12**	**XX (Full)**	**4-5**	**3-5**	**132K/cm²**	**6**
			F7L2	F7C1	P12					
			F7L3	F7C3	P12					
**M4**	**M**	**21**	**M4L1**	**M4C8**	**P14**	**XY (Full)**	**4-5**	**3-4**	**132K/cm²**	**7**
			M4L2	M4C5	P19					
			M4L3	M4C3	P14					
**M7**	**M**	**21**	**M7L1**	**M7C16**	**P8**	**XY (QC)**	**4-5**	**3-4**	**132K/cm²**	**7**
			M7L2	M7C17	P8					
			M7L3	M7C2	P8					
**M9**	**M**	**27**	**M9L1**	**M9C10**	**P8**	**XY (QC)**	**5-6**	**4-5**	**197K/cm²**	**4**
			M9L2	M9C5	P8					
			M9L3	M9C6	P8					

Table S3. Antibody dilution, supplier and catalog number.

Target	Dilution	Isotype	Conjugate	Assay	Supplier	Catalog #
cTnT	1:400	Mouse IgG1	None	FC	Thermo Fisher	MS-295-P1
Isotype	1:100	Mouse IgGK	FITC	FC	Thermo Fisher	11-4714-81
Isotype	1:25	Mouse IgG3	DL-650	FC	Thermo Fisher	MA1-190-D650
Mouse IgG	1:250	Goat	AF-488	IHC	Thermo Fisher	A11001
Mouse IgG	1:400	Goat	AF-647	FC	Thermo Fisher	A21235
Mouse IgM	1:250	Goat	AF-555	IHC	Thermo Fisher	A24871
Oct4	1:200	Rabbit IgG	None	IHC	Thermo Fisher	A24867
Oct4	1:100	Mouse IgGK	FITC	FC	Thermo Fisher	53-5841-82
Rabbit IgG	1:250	Donkey	AF-549	IHC	Thermo Fisher	A24870
Rat	1:250	Donkey	AF-488	IHC	Thermo Fisher	A2487
Sox2	1:100	Rat	None	IHC	Thermo Fisher	A24759
SSEA4	1:100	Mouse IgG3	None	IHC	Thermo Fisher	A24866
SSEA4	1:25	Mouse IgG3	AF-647	FC	Thermo Fisher	SSEA421
TRA-1-60	1:100	Mouse IgM	None	IHC	Thermo Fisher	A24868

Table S4. Forward and reverse primer sequences for q-RT-PCR.

Gene	Accession Number	Forward Primer	T_m (°C)	Reverse Primer	T_m (°C)
CDH7	NM_033646	GAGCCATCCAATGGACACCAGT	59.5	GAAGCCGTTTCTCCTGGTCAGT	59.2
CNTN4	NM_175607	GGACCACTTTGAAAGAGTTGGAG	55.8	CAGCAGATAGCCTGTCCACACT	58.9
EDF-1	NM_031886	TCGAGACGCTGCCTGACTTCC	61.4	CACCACGAAGAACGGGTCATT	57.6
KCNA7	NM_031886	TCGAGACGCTGCCTGACTTCC	61.4	CACCACGAAGAACGGGTCATT	57.6
KCNE1	NM_000219	TCTACGTCCTCATGGTACTGG	56.2	TGTCCTTCTCTTGCCAGGCAT	59.4
KCNJ12	NM_021012	CCAGTGCAACATTGAGTTCGCC	59.2	AGCGAGAAGATGAGCAGCATG	58.3
NANOG	NM_001297698.1	CTCCAACATCCTGAACCTCAGCT	61	CACACCATTGCTATTCTTCGGCC	61

NR4A2	NM_006186	AAACTGCCCAGTGGACAAGCG	60.6	GCTCTTCGGTTTCGAGGGCAA	60.1
OCT4	(POU5F1)	CCTGAAGCAGAAGAGGATCACCC	61	AAAGCGGCAGATGGTCGTTTG	61
SCN3B	NM_018400	TCTGGTCTTCCTCACCTTGTGG	58.6	TGGGATGGCAAGGTAGTCAGAC	58.7
Sendai Virus (SeV)	NA (Thermo Fisher Catalog)	GGATCACTAGGTGATATCGAGC	55	ACCAGACAAGAGTTTAAGAGATATGTATC	55
SOX2	(NM_003106)	AGCTACAGCATGATGCAGGACC	61	CGAGCTGGTCATGGAGTTGTAC	60
TRH	NM_007117	CCTGGATGACTTCCTGCGCCA	62	TTGCCTGGATGCTGACGTTTGG	60.4

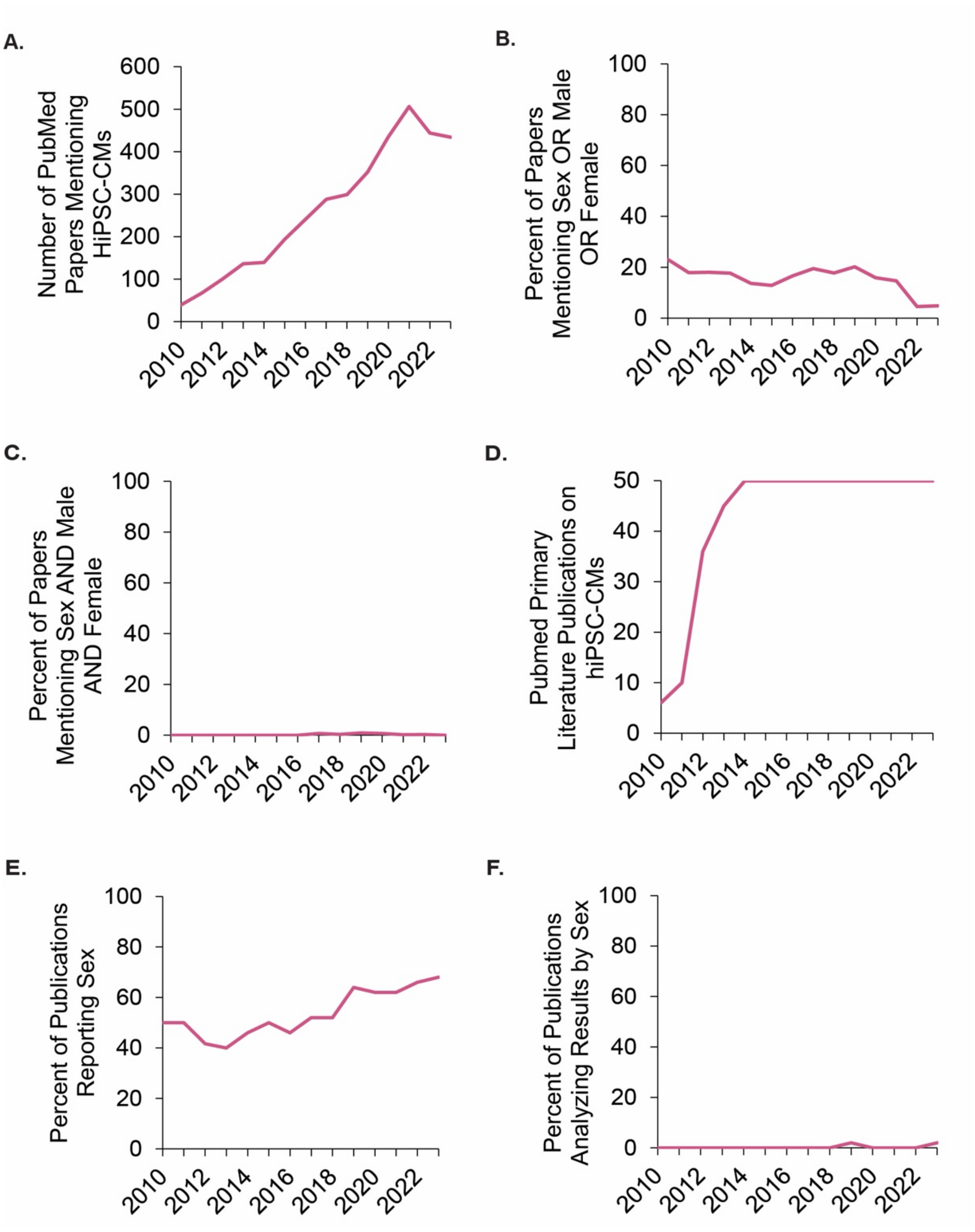
A.
Number of PubMed Papers Mentioning HiPSC-CMs
600
500
400
300
200
100
0
2010
2012
2014
2016
2018
2020
2022
B.
Percent of Papers Mentioning Sex OR Male OR Female
100
80
60
40
20
0
C.
Percent of Papers Mentioning Sex AND Male AND Female
D.
Pubmed Primary Literature Publications on hiPSC-CMs
50
40
30
20
10
E.
Percent of Publications Reporting Sex
F.
Percent of Publications Analyzing Results by Sex

Figure S1. Sex is underreported and understudied in hiPSC-CM literature.

A) A line graph illustrating the annual number of papers retrieved when searching "(Human Induced Pluripotent Stem Cell derived Cardiomyocytes) OR hiPSC-CMs OR (human iPSC-CMs)" on PubMed for the years 2010 to 2023. B) A line graph illustrating the annual number of papers retrieved when searching "((Human Induced Pluripotent Stem Cell derived Cardiomyocytes) OR hiPSC-CM OR (human iPSC-CMs)) AND (Sex OR Male OR Female)" in PubMed for the years 2010 to 2023. C) A line graph illustrating the annual number of papers retrieved when searching "((Human Induced Pluripotent Stem Cell derived Cardiomyocytes) OR hiPSC-CMs OR (human iPSC-CMs)) AND (Sex AND Male AND Female)" on PubMed for the years 2010 to 2023. D) A line graph displaying the number of papers more thoroughly assessed in (E) and (F) when searching "(Human Induced Pluripotent Stem Cell derived Cardiomyocytes) OR hiPSC-CMs OR (human iPSC-CMs)" on PubMed for the years 2010 to 2023. E) Represents the number of primary literature papers that reported the sex of the hiPSC-line and F) displays the number of primary literature papers that analyzed their results by sex when searching the query above on PubMed.

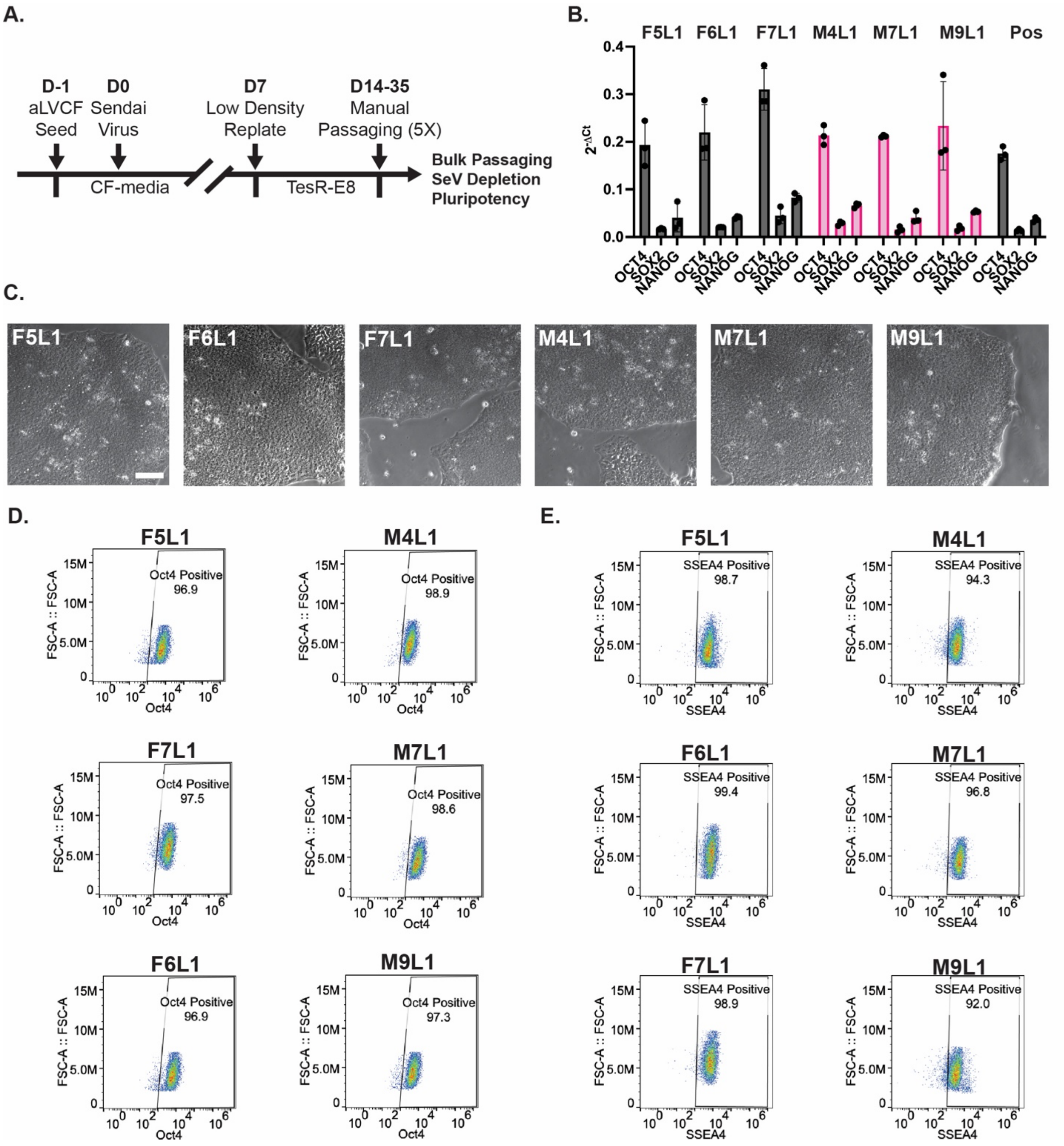

A.
D-1 aLVCF Seed
D0 Sendai Virus
D7 Low Density Replate
D14-35 Manual Passaging (5X)
CF-media
TesR-E8
Bulk Passaging
SeV Depletion
Pluripotency
B.
F5L1 F6L1 F7L1 M4L1 M7L1 M9L1 Pos
2^-ΔCt
OCT4 SOX2 NANOG
C.
F5L1 F6L1 F7L1 M4L1 M7L1 M9L1
D.
F5L1 Oct4 Positive 96.9
M4L1 Oct4 Positive 98.9
F7L1 Oct4 Positive 97.5
M7L1 Oct4 Positive 98.6
F6L1 Oct4 Positive 96.9
M9L1 Oct4 Positive 97.3
FSC-A :: FSC-A
Oct4
E.
F5L1 SSEA4 Positive 98.7
M4L1 SSEA4 Positive 94.3
F6L1 SSEA4 Positive 99.4
M7L1 SSEA4 Positive 96.8
F7L1 SSEA4 Positive 98.9
M9L1 SSEA4 Positive 92.0
SSEA4

Figure S2. Adult left ventricular cardiac fibroblasts reprogramming into hiPSCs.
A) Schematic depicting the Cyto-Tune 2.0 Sendai virus hiPSC reprogramming protocol used in this study. B) Pluripotency validation via q-RT-PCR of OCT4, SOX2, and NANOG as compared to a previously established line as a positive control (Pos). C) Brightfield images showing the characteristic hiPSC colony morphology in the adult left ventricular derived-hiPSCs from all 6 donors. Flow cytometry of the pluripotency marker D) Oct4 and E) SSEA4 for all 6 donors with the gate decided based on an isotype-control. Scale 50µm. For (B) the bar and error bars represent the mean ± STDEV and each dot represents one technical replicate for one passage of each hiPSC-lines after Sendai virus depletion where a line is defined as Sendai virus depleted when there is no amplification of q-RT-PCR Sendai virus primers for two passages.

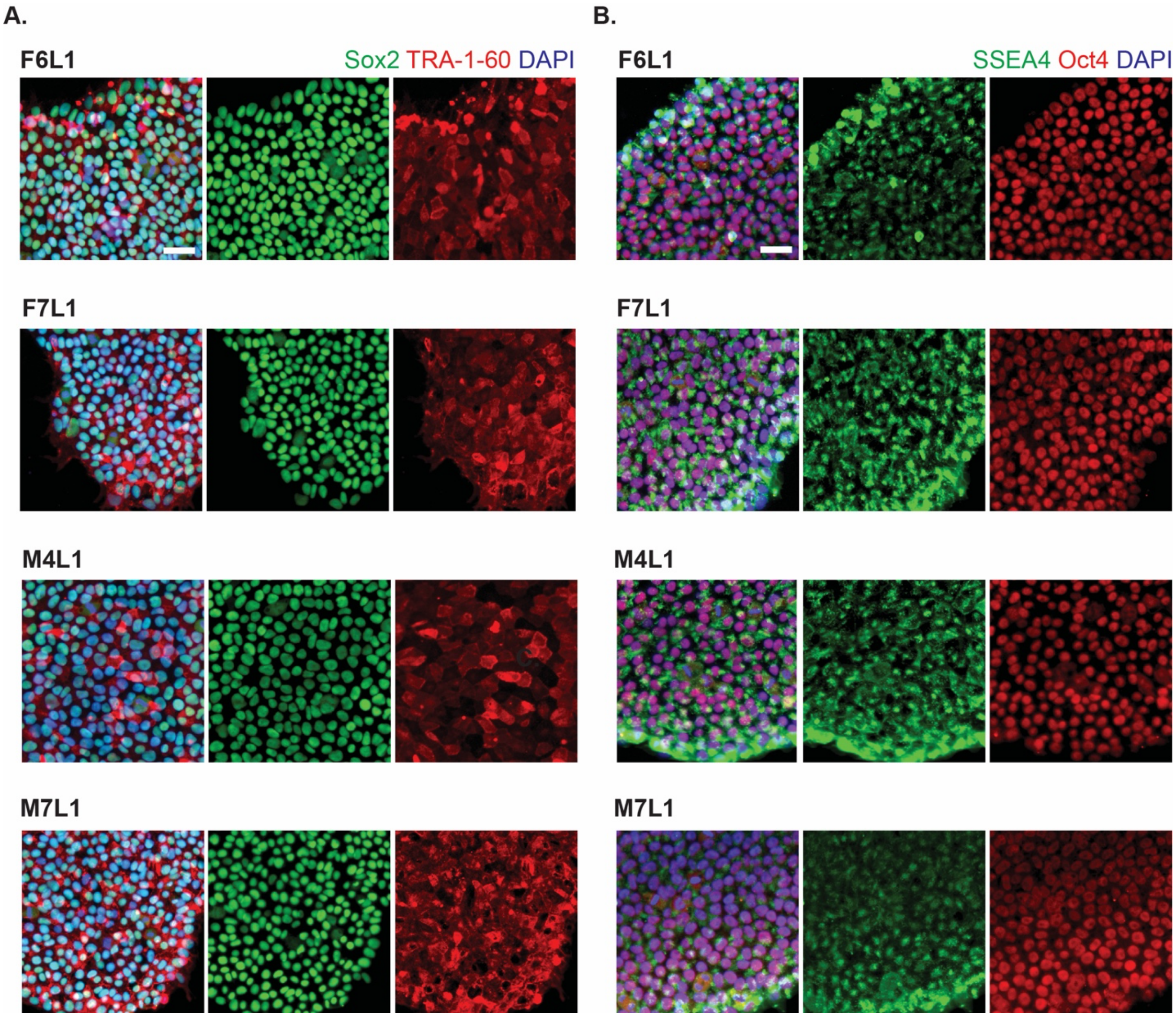

Figure S3. Extended pluripotency validation.

A) Representative IHC from F6L1, F7L1, M4L1 and M7L1 of the nuclear protein, Sox2 (green), and the surface protein TRA-1-60 (red) as well as a merged image with the nuclear marker DAPI (blue). C) Representative IHC from F6L1, F7L1, M4L1, and M7L1 of the surface protein SSEA4 (green) and the nuclear protein Oct4 (red) as well as a merged image with the nuclear marker DAPI (blue). Scale 50 μm.

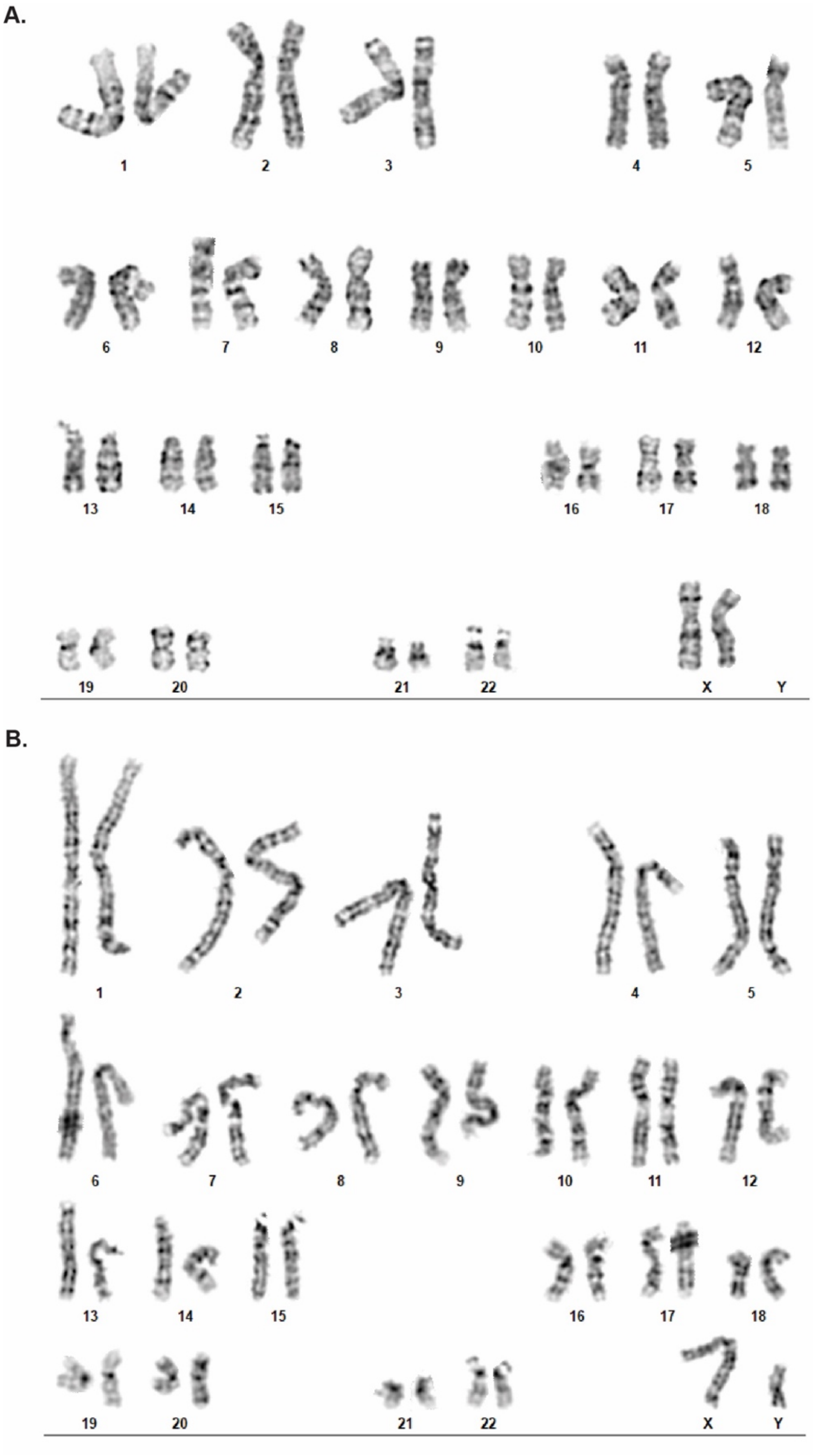

Figure S4. Full Karyotyping.

Full karyotype for A) F7L1 with a XX karyotype and B) M4L1 with an XY karyotype showing no chromosomal abnormalities.

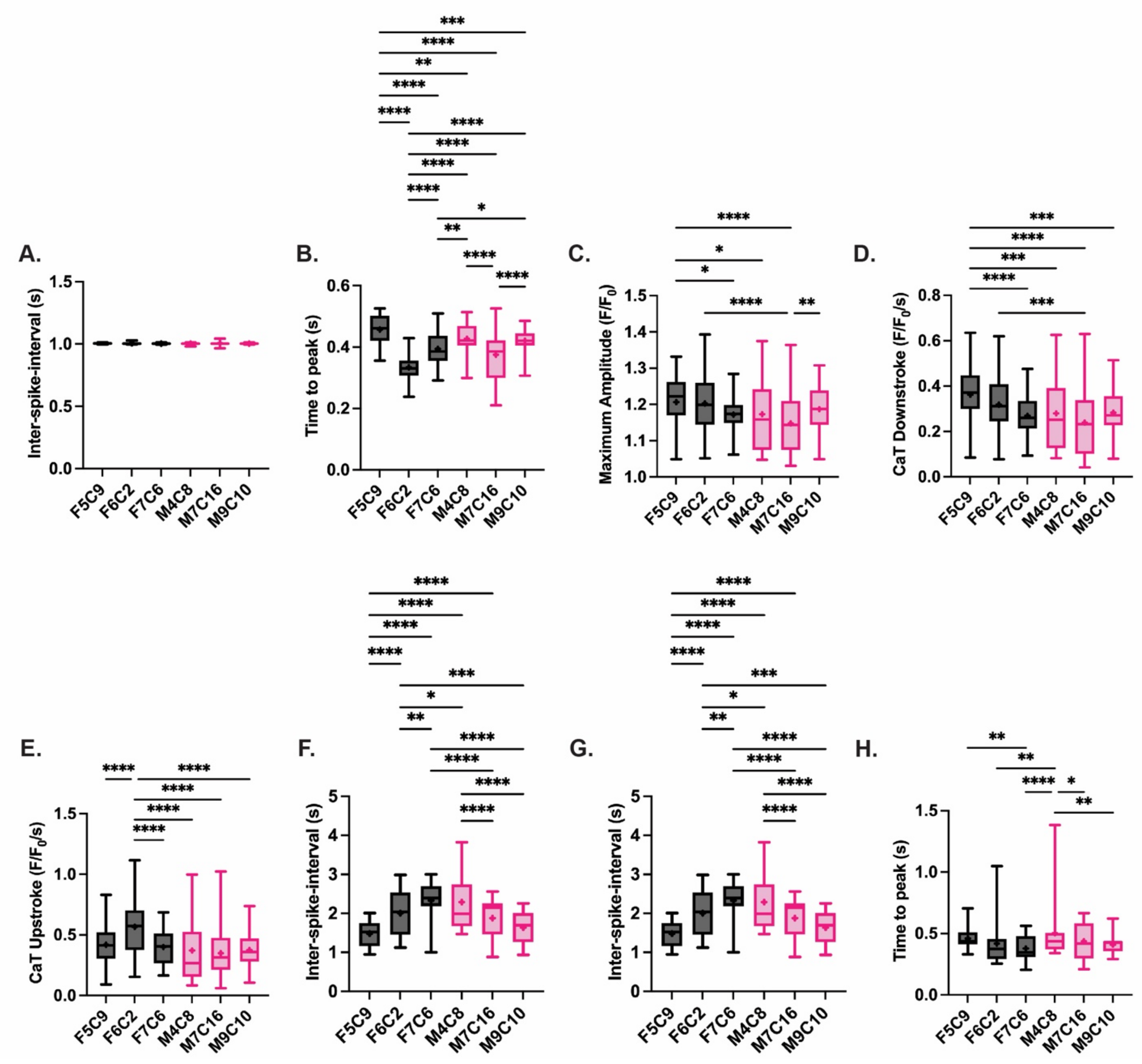

Figure S5. Calcium transient analysis by line.

The paced calcium transients (CaT) data by line showing box and whisker plots of the A) Inter-spike-interval (ISI), B) time to peak, C) maximum amplitude, and the CaT maximum D) downstroke and E) upstroke velocity. The spontaneous calcium transient data showing the F) ISI, G) the coefficient of variance of the ISI as an assessment of beating irregularity and the H) time to peak. Statistics were conducted using a one-way ANOVA Tukey HSD comparison of means. Where $*p < 0.05$, $**p < 0.01$, $***p < 0.001$, and $****p < 0.0001$.

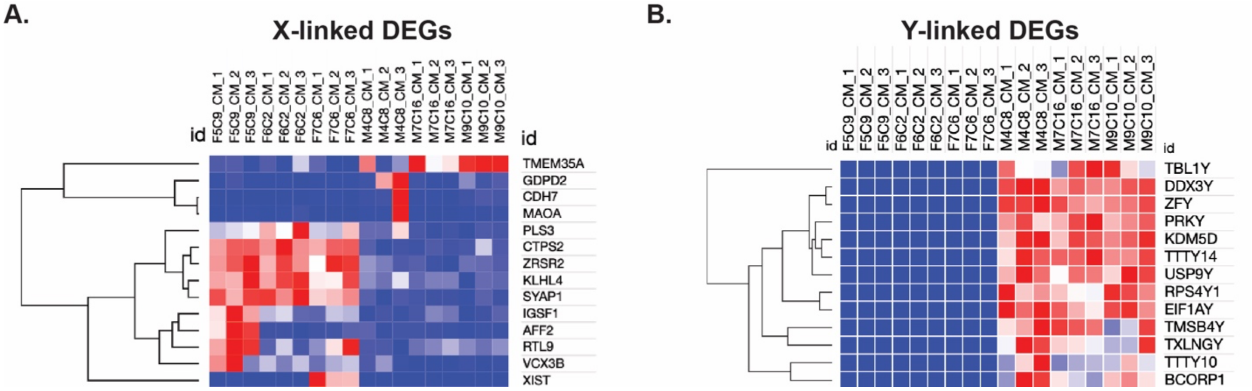

Figure S6. Bulk RNA sequencing sex chromosome-linked DEGs. Heat maps of DEGs located on the A) X and B) Y chromosomes.

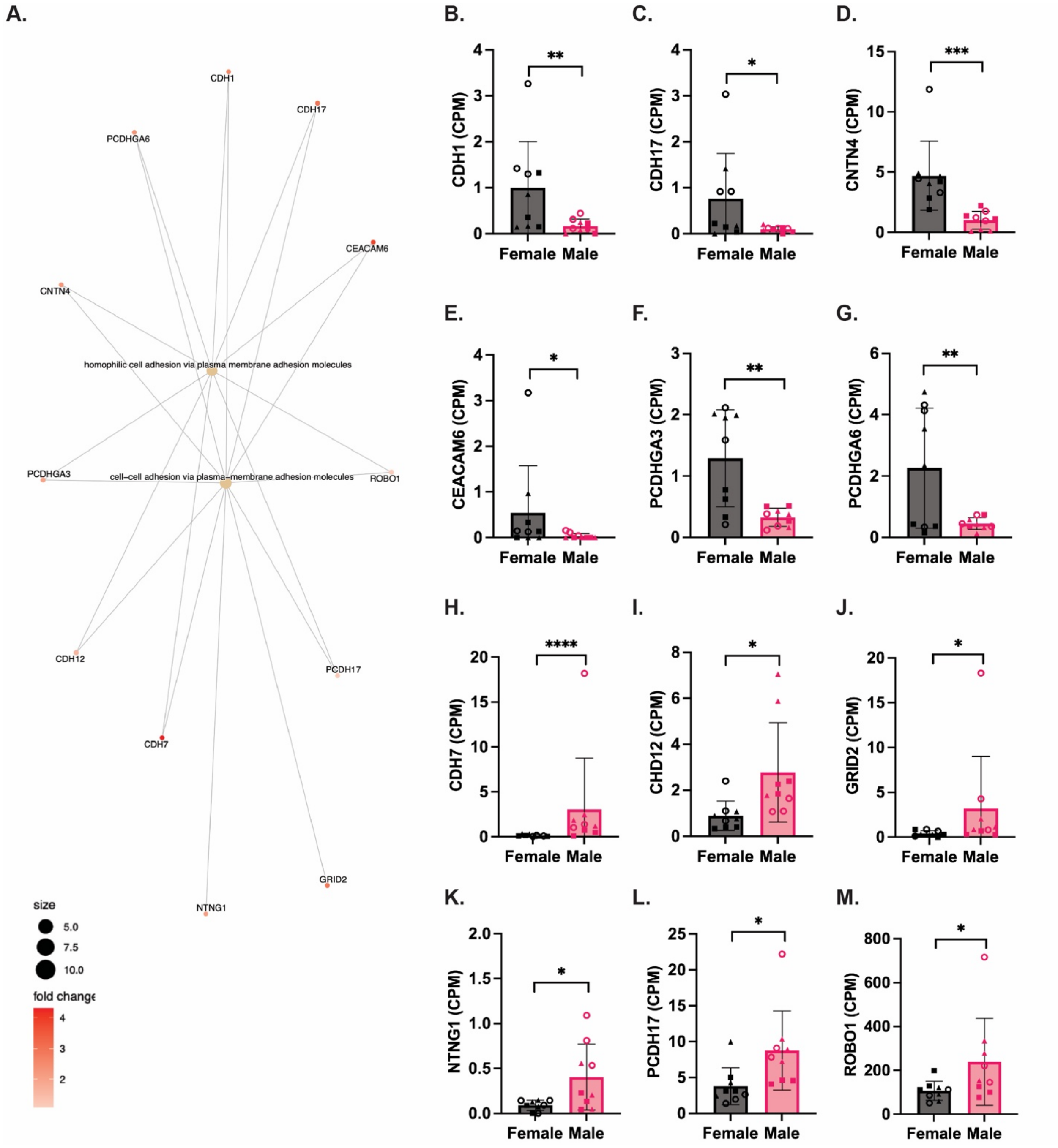

A.
CDH1
CDH17
PCDHGA6
CEACAM6
CNTN4
homophilic cell adhesion via plasma membrane adhesion molecules
PCDHGA3
cell-cell adhesion via plasma-membrane adhesion molecules
ROBO1
CDH12
PCDH17
CDH7
GRID2
NTNG1
size
5.0
7.5
10.0
fold change
4
3
2
B.
CDH1 (CPM)
C.
CDH17 (CPM)
D.
CNTN4 (CPM)
E.
CEACAM6 (CPM)
F.
PCDHGA3 (CPM)
G.
PCDHGA6 (CPM)
H.
CDH7 (CPM)
I.
CHD12 (CPM)
J.
GRID2 (CPM)
K.
NTNG1 (CPM)
L.
PCDH17 (CPM)
M.
ROBO1 (CPM)
Female Male

Figure S7. Computer network (cnet) of genes in the enriched cell-cell adhesion gene ontology (GO) pathways.

A) The cnet plot showing the differentially expressed genes that caused enrichment of the gene ontology GO pathways "homophilic cell adhesion via plasma membrane adhesion molecules" and "cell-cell adhesion via plasma membrane adhesion molecules." (B-M) The average counts per million for each gene that is enriched in the pathways on the cnet plot. For (A) The color of each gene indicates the gene's fold change, reflecting its differential expression between male and female-derived cardiomyocytes (CMs), as detailed in the legend on the left-hand side of the figure. For (B-M) the bar represents the average ± STDEV and each dot is the expression level for one independent experiment with n = 3 replicates per line and three hiPSC-CM lines per sex. Where O – F5L1 and M4L1, ■ – F6L1 and M7L1 and ▲ – F7L1 and M9L1. Statistical significance designations are based on an Edge Test of raw reads and the False Discovery Rate corrected p-value. The test that was used to determine the DEGs. Where *$p < 0.05$, **$p < 0.01$, ***$p < 0.001$, and ****$p < 0.0001$.

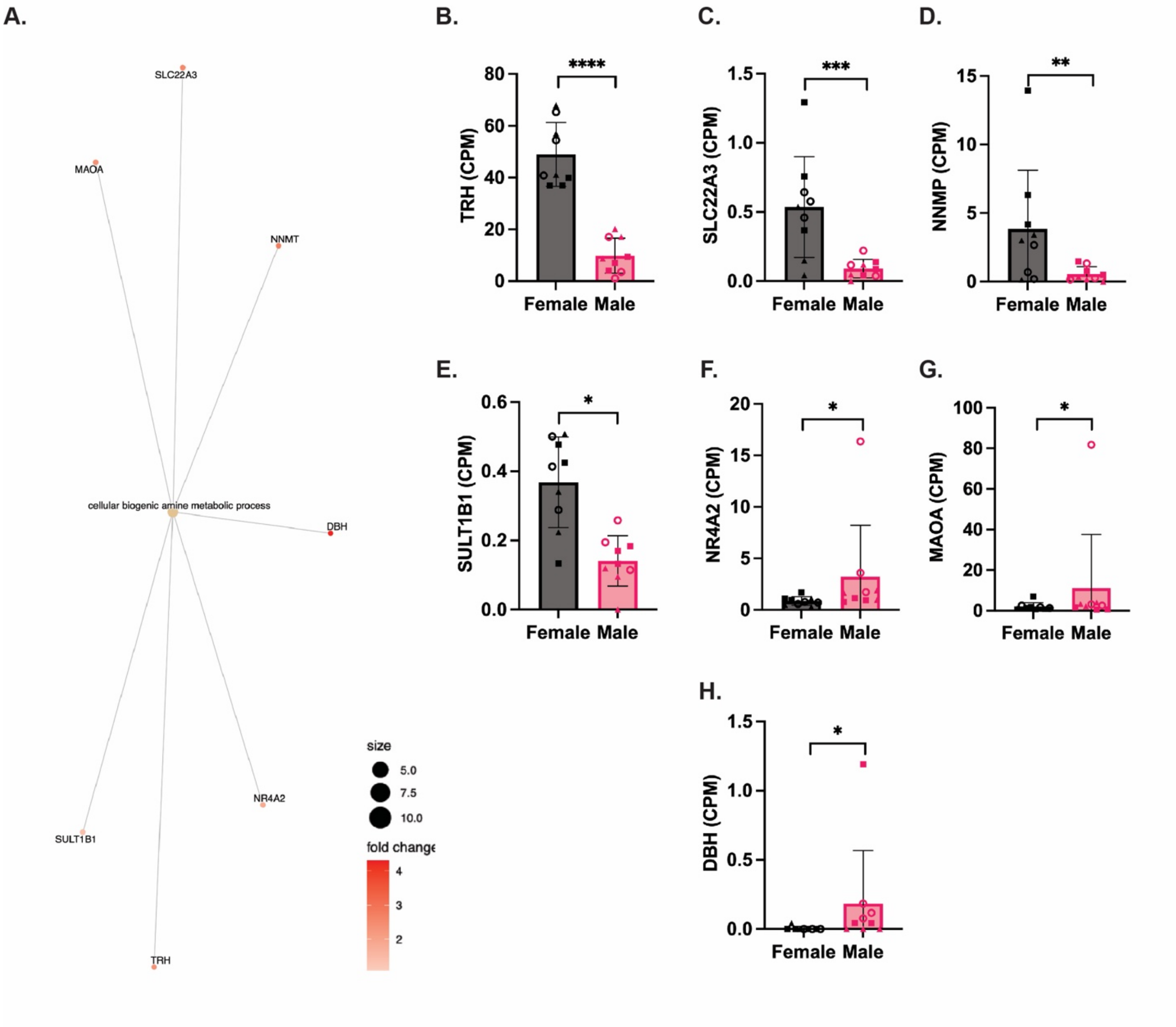
A.
SLC22A3
MAOA
NNMT
cellular biogenic amine metabolic process
DBH
NR4A2
SULT1B1
TRH
size
5.0
7.5
10.0
fold change
4
3
2
B.
TRH (CPM)
****
Female Male
C.
SLC22A3 (CPM)
***
Female Male
D.
NNMP (CPM)
**
Female Male
E.
SULT1B1 (CPM)
*
Female Male
F.
NR4A2 (CPM)
*
Female Male
G.
MAOA (CPM)
*
Female Male
H.
DBH (CPM)
*
Female Male

Figure S8. Computer network (cnet) of genes in the enriched amine metabolic process gene ontology (GO) pathway.

A) The cnet plot showing the differentially expressed genes that caused the enrichment of the GO pathway "cellular biogenic amine metabolic process." (B-H) The average counts per million for each gene that is enriched in the pathway. For (A) The color of each gene indicates the gene's fold change, reflecting its differential expression between male and female-derived cardiomyocytes (CMs), as detailed in the legend on the left-hand side of the figure. For (B-M) the bar represents the average ± STDEV and each dot is the expression level for one independent experiment with n = 3 replicates per line and three hiPSC-CM lines per sex. Where O – F5L1 and M4L1, ■ – F6L1 and M7L1 and ▲ – F7L1 and M9L1. Statistical significance designations are based on an Edge Test of raw reads and the False Discovery Rate corrected p-value. The test that was used to determine the DEGs. Where *p < 0.05, **p < 0.01, ***p < 0.001, and ****p < 0.0001.

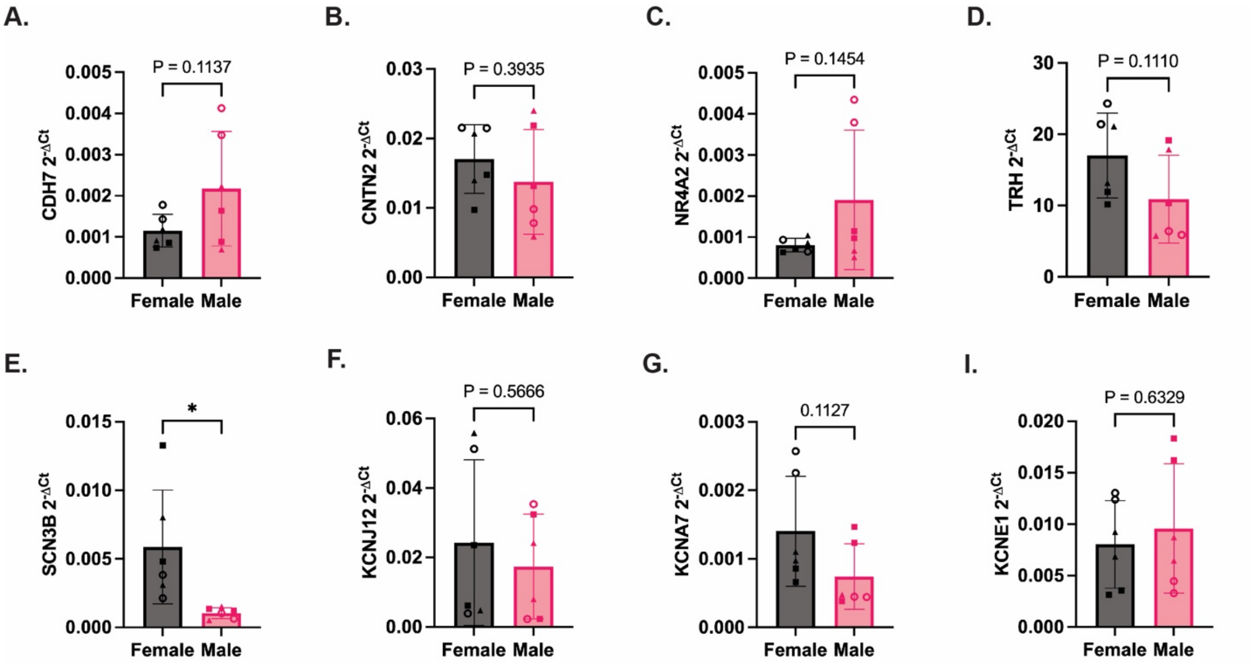

Figure S9. q-RT-PCR validation of bulk RNA sequencing data.

Validation of the gene expression using q-RT-PCR for genes enriched in the cell-cell adhesion pathway A) CDH7 and B) CNTN2, genes enriched in the amine metabolic process pathway C) NR4A2 and D) TRH as well as ion channel associated genes E) SCN3B, F) KCNJ12, G) KCNA7 and I) KCNE1. For A-I) each dot represents the average $2^{-\Delta Ct}$ of three technical replicates normalized to the housekeeping gene EDF1. Two batches of hiPSC-CM per line were assessed with each line being defined as O – F5L1 and M4L1, ■ – F6L1 and M7L1 and ▲ – F7L1 and M9L1. Statistical comparisons were made using a pairwise Student's t-test with *p < 0.05, **p < 0.01, ***p < 0.001, and ****p < 0.0001.

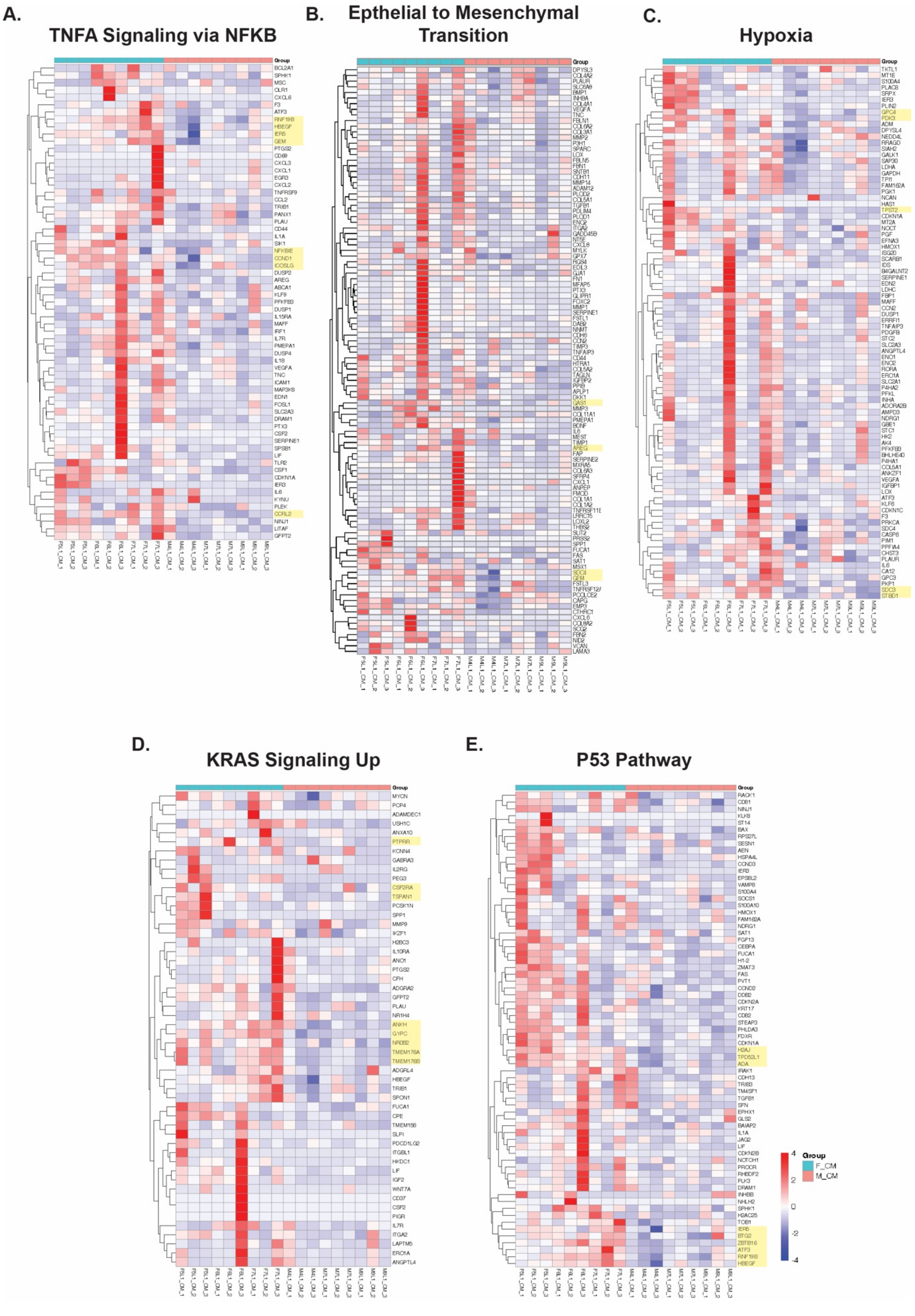
A.
TNFA Signaling via NFKB
B.
Epthelial to Mesenchymal Transition
C.
Hypoxia
D.
KRAS Signaling Up
E.
P53 Pathway
Group
F_CM
M_CM

Figure S10. Enriched hallmark gene sets showing the genes responsible for suppression in male hiPSC-CM.

Heatmaps of the genes that resulted in enrichment of the hallmark gene sets for A) hypoxia, B) P53 pathway, A) TNFA signaling via NFKB, B) epithelial to mesenchymal transition, C) hypoxia, D) KRAS signaling up, and B) P53 pathway. Each square represents the counts per million of three independent experiments from each of the lines. Genes that showed consistent trends across all lines were highlighted.
